# A Survey on Computational Methods in Drug Discovery for Neurodegenerative Diseases

**DOI:** 10.3390/biom14101330

**Published:** 2024-10-19

**Authors:** Caterina Vicidomini, Francesco Fontanella, Tiziana D’Alessandro, Giovanni N. Roviello

**Affiliations:** 1 Institute of Biostructures and Bioimaging-Italian National Council for Research (IBB-CNR), Via De Amicis 95, 80145 Naples, Italy; 2 Department of Electrical and Information Engineering “Maurizio Scarano”, University of Cassino and Southern Lazio, 03043 Cassino, Italy

**Keywords:** neurodegeneration, neurodrugs, machine learning, molecular docking, artificial intelligence, drug discovery, Alzheimer’s disease, Parkinson’s diseases

## Abstract

Currently, the age structure of the world population is changing due to declining birth rates and increasing life expectancy. As a result, physicians worldwide have to treat an increasing number of age-related diseases, of which neurological disorders represent a significant part. In this context, there is an urgent need to discover new therapeutic approaches to counteract the effects of neurodegeneration on human health, and computational science can be of pivotal importance for more effective neurodrug discovery. The knowledge of the molecular structure of the receptors and other biomolecules involved in neurological pathogenesis facilitates the design of new molecules as potential drugs to be used in the fight against diseases of high social relevance such as dementia, Alzheimer’s disease (AD) and Parkinson’s disease (PD), to cite only a few. However, the absence of comprehensive guidelines regarding the strengths and weaknesses of alternative approaches creates a fragmented and disconnected field, resulting in missed opportunities to enhance performance and achieve successful applications. This review aims to summarize some of the most innovative strategies based on computational methods used for neurodrug development. In particular, recent applications and the state-of-the-art of molecular docking and artificial intelligence for ligand- and target-based approaches in novel drug design were reviewed, highlighting the crucial role of in silico methods in the context of neurodrug discovery for neurodegenerative diseases.

## 1. Introduction

Age-related diseases have heavy economic and psychological effects on society. Neurodegenerative diseases, including Parkinson’s disease, Alzheimer’s disease, multiple sclerosis and motor neuron disease, are among the most prevalent and high-cost to treat. Therefore, research into effective therapeutics to overcome these socially relevant pathologies is urgently needed. In this regard, various families of compounds have been found to possess neuroprotective properties or could be chemically modified to develop more effective neurodrugs. For example, natural products and their derivatives are gaining increasing attention in drug discovery programs that have identified microorganisms that produce a wide range of metabolites with bioactive properties [1]. Great efforts were made in order to develop efficacious therapies for nervous system disorders and such approaches were based on the identification of the main biomolecules that have important roles in the central nervous system (CNS). However, most screenings are typically conducted randomly rather than systematically. The discovery of new molecules that target the CNS was the subject of several studies with potential therapeutic applications starting in the 19th century [2] and still continuing [3,4]. Remarkably, the first drugs used to treat central nervous system diseases were based on natural resources, especially plants. However, studies targeting plants with this type of bioactivity represent only a tiny fraction of the investigations performed in neurodrug discovery. Several specis were the subject of accurate study and, for the leading plant families, some molecular mechanisms behind the biological activity concerning the nervous system and the active substances that support this activity were identified [2]. In recent decades, the emergence of an interdisciplinary fusion of sciences in the form of “bioinformatics” has significantly impacted the drug development process, including neurodrug development. Famously, the applicability of bioinformatics approaches to pharmaceutical research can reduce overall costs and significantly abbreviate times needed for drug discovery and development [5]. Recognized as a commercially viable solution to this problem, thanks to the broad applicability of the Computer-Aided Drug Discovery System (CADDS), bioinformatics methods have been widely employed to perform high-throughput primary virtual screening. The function of various bioinformatics tools for virtual screening and the molecular dynamics of molecules/drugs selected from herbs, as well as semi-synthetic and synthetic compounds, was demonstrated to help predict potential therapeutic interventions for neurodegeneration [6]. Artificial intelligence, abbreviated as AI, and especially its subcategory known as machine learning (ML), discloses enormous possibilities for modern neurodrug discovery and effective therapy development [7]. A significant number of machine learning methodologies have been reported in the scientific literature recently, and among such processes many of the examples are of domain-specific AI that were applied successfully to drug discovery and design [5]. Another in silico method of fundamental importance to aid modern neurodrug discovery is molecular docking. This is an established structure-based computational technique that finds use largely in the screening process of large numbers of molecular structures to be used in biomedicine. Docking can lead to the identification of new compounds of pharmaceutic interest and very importantly, allows one to predict the key ligand–target interactions at the molecular level and to describe the main structure–activity relationships (SARs) that can be applied in lieu of identifying the chemical structures of other target biological modulators a priori [8]. Although molecular docking was used initially for the comprehension of the mechanisms of biomolecular recognition taking place between small ligands and biomacromolecules of a different nature, recently, the use and application of docking in drug (including neurodrug) discovery have evolved remarkably, opening new routes to discover efficacious neurodrugs. However, this field still needs a comprehensive survey on state-of-the-art molecular docking despite the increasing interest in using computational methods for drug discovery. This lack results in the missing of opportunities to enhance the performance of these promising methods and implement successful applications. This lack is even more problematic for neurodegenerative diseases, which present a worldwide social emergency because of their ever-increasing incidence. This paper tries to fill this gap by presenting a review of innovative strategies based on computational methods applicable to neurodisease therapy and, in particular, we reviewed recent avenues in molecular docking and artificial intelligence science, highlighting the key role of in silico processes in the context of ligand- and target-based neurodrug discovery. The remainder of this paper is organized as follows. Section 2 describes the background of neurodegenerative diseases. Section 3 discusses molecular docking and neurodrug development approaches. Section 4 presents recent machine learning techniques for drug discovery, whereas Section 5 provides an overview of the use of these techniques for neurodrug discovery. Finally, conclusions are eventually shown in Section 6.

## 2. Neurodegenerative Diseases

Human life expectancy has increased in the last decades, and consequently, the population worldwide has greatly increased. At the same time, this has led to increased numbers of individuals affected by neurodegenerative diseases (NDDs) [9]. Neuropathologies may originate as a consequence of biological events that can affect both the CNS and Peripheral Nervous Systems (PNS), when the core communication circuitry is compromised by the loss of neurons and, thus, both the structure and related functions that normally exist in these neural networks collapse. Notoriously, the neurons cannot effectively renew themselves because of their specific characteristics of terminal differentiation. Hence, this results in impaired memory, behavior, sensory processing, cognition, and motor function following neurodegeneration. Together with infectious diseases, NDDs are the most challenging pathologies, requiring the urgent development of cutting-edge drugs. For example, dementia is one of the main causes of disability and dependency among older people worldwide and is currently the seventh highest cause of death among all diseases. Dementia affects, not only patients psychologically, physically, economically and socially, but also affects their caregivers and society, representing one of the primary causes of disability and dependency among older people. One of the main problems is that dementia is frequently misunderstood, representing just the tip of the iceberg in many different forms of NDDs, which leads to stigmatization and obstacles to diagnosis and care [10]. 

The most prevalent form of dementia is caused by Alzheimer’s Disease (AD) [11,12], which is responsible for 60–70% of cases and, together with Parkinson’s Disease (PD) [13], today acts as the most important and widespread neurodegenerative disease [14]. AD presents different forms that can be classified as familial and sporadic [15]. 

Aging is a well-established risk factor for neurodegenerative diseases such as dementia and AD, making it a promising area for identifying novel therapeutic targets. Research has focused on how age-related molecular changes may contribute to dementia, with particular attention given to declining NAD+ (Nicotinamide adenine dinucleotide) levels, a critical coenzyme involved in cellular metabolism and DNA repair. The coenzyme NAD+ plays a vital role in cellular energy production and stress response. Its decline is a hallmark of aging, potentially contributing to various chronic conditions. Maintaining adequate NAD+ levels is particularly crucial for high-energy-demanding cells and proper neuronal function. Reduced NAD+ levels have been observed in major neurodegenerative conditions like AD and PD, as well as in cardiovascular diseases and muscle atrophy. Recent studies indicate that NAD+ depletion occurs across multiple tissues during aging. Moreover, both physiological and pharmacological strategies to boost NAD+ levels show promise in slowing down aspects of aging and preventing certain age-related diseases [16]. Moreover, recent studies suggest that analyzing plasma proteins in middle-aged adults can help predict dementia risk decades before symptoms appear. A large-scale proteomics study examined 4877 plasma proteins in over 10,000 middle-aged individuals and identified 32 proteins associated with a 25-year risk of developing dementia [17]. These proteins are linked to key biological processes [18,19,20], including immune response, protein regulation and brain synaptic functions. Further analyses confirmed that some of these proteins correlated with Alzheimer’s-related biomarkers in cerebrospinal fluid and were abnormally expressed in the brain tissues of AD patients. Notably, disruptions in immune and protein regulation pathways appeared up to 20 years before the dementia onset, and abnormalities in the coagulation pathways emerged about 10 years prior. This study also used genomics methods to validate several of these proteins as markers of AD, identifying SERPINA3 as a potential driver of the disease [18,19,20]. These findings suggest that specific plasma proteins could serve as early indicators of dementia risk, offering a window for earlier intervention. This is particularly relevant when considering the familial form of AD, which is an autosomal dominant disease characterized by early onset (EOAD) and affects people under 65 (one to five percent of cases). It is characterized by changes in specific genes, such as the presenilin 1 gene (PSEN1), linked to familial AD in up to 70% of cases, the presenilin 2 gene (PSEN2) and the amyloid precursor protein gene (APP). On the other hand, the late-onset sporadic presentation of AD (LOAD) affects mainly people over 65. As for sporadic AD, the primary risk factor is thought to be ageing [21,22], but since it represents a complex disorder, other risk factors have also been identified, including traumatic brain injury, female sex, environmental pollution, depression, social isolation, low academic achievement, physical inactivity, metabolic syndrome [10,21] and genetic susceptibility. Particularly, mutations in the 4 allele of apolipoprotein E (APOE), which has a heritability of up to 60–80%, are common in AD. 

Notably, recent studies highlight a significant co-morbidity between incidence of type 2 diabetes mellitus (T2DM) and neurodegeneration, revealing shared molecular pathways that underlie both disease processes [23,24]. A key factor contributing to this link is mitochondrial dysfunction, which is prominently observed in the early stages of both T2DM and neurodegenerative disorders. Mitochondria play a crucial role in glucose metabolism, positioning them as vital regulators of various cellular processes impacted in these diseases [25]. A deeper understanding of mitochondrial involvement in the pathogenesis of T2DM and neurodegeneration could pave the way for new pharmacological therapies aimed at restoring mitochondrial function, ultimately enhancing health span and improving treatment outcomes for both conditions.

### 2.1. Hallmarks in NDDS 

To manage neurological diseases, it is essential to identify trustworthy hallmarks to enhance differential diagnosis, which is crucial for prognostic purposes, as well as to identify new biomolecular targets for drug discovery and the prediction of therapeutic response [26]. The hallmarks, i.e., the biological and genetic evidence and the specifically linked biomarkers let us understand and monitor the “real nature” of diseases, revealing that what we had so far considered in an extremely simplistic way instead is the result of multifactorial components [27]. To better understand the connections between human biology and disease phenotype, it is essential to highlight the connections between the underlying molecular and cellular processes and the use of biomarkers in vivo, identifying and tracking such specific hallmarks [27,28,29]. Genetic variables and metabolic pathways underpinning numerous NDDs have been uncovered through decades of basic, translational and clinical research, leading to the discovery of eight NDDs hallmarks that can be listed as reported below: pathological protein aggregation, neuronal and synaptic network dysfunction [30,31], aberrant proteostasis [32,33], cytoskeletal abnormalities [34,35], altered energy metabolism [36,37], RNA and DNA defects [38,39], inflammatory states [40,41] and neuronal cell death [42]. These hallmarks are listed in the context of prevalent NDDs, including dementia, as Frontotemporal dementia (FTD) and AD, synucleinopathies (i.e., multisystem atrophy [MSA] and Lewy body dementia [LBD]), primary tauopathies, PD, amyotrophy lateral sclerosis (ALS), prion disease (PrD), Huntington disease (HD) and related polyglutamine (polyQ) diseases (including spinocerebellar ataxias [SCA]), multiple sclerosis (MS), chronic traumatic encephalopathy (CTE), traumatic brain injury (TBI), spinal cord injury (SCI) and stroke. As for the development of abnormal protein conformations, the critical aggregation step has molecular roots in mutations, truncations and posttranslational changes. Amyloid beta (Aβ) peptide and tau protein in AD, tau protein in primary tauopathies (including progressive supranuclear palsy [PSP]) and α-synuclein in PD and other synucleinopathies, are some of the main proteins implicated in this aggregative mechanism at the origin of the neurodegenerative processes. The discovery of causal mutations responsible for pathogenic pathways of inherited forms of NDDs opened the way for new investigations on NDDs’ pathogenic processes. In fact, several NDDs share a common trait linking protein aggregation with disease-causing mutations. Many studies support the hypothesis that aggregation leads to a toxic gain-of-function mechanism rather than a loss in function of the protein encoded by the involved genes [43]. In this context, Aβ, a small peptide consisting of 39 to 43 amino acids, is regarded as the primary component of neocortical plaques in CNS, which serves as the pathological hallmark of AD. [44,45,46]. The APP gene could present many mutational patterns that were remarkable in their production of Aβ peptide, [47]. The APP gene, in fact, has a total of 17 exons and produces several isoforms through the alternative exon splicing of exons 7 and 8 [48]. Among them, three isoforms, the 695, 751 and 770, are important in AD and are expressed in the central nervous system [49]. At the end of the sequence, two exons, 16 and 17, encode the APP section that becomes the Aβ fragment after proteolytic processing [50]. Here, we find the region of the APP gene that codes for the proteolytic sites of β- and γ-secretases that cause an overall rise in the production of Aβ [51]. The products generated by the APP mutations can exhibit an increase of 42 to 40 amino acid-long Aβ (Aβ 42/ Aβ 40) ratios and an increase in total tau and phosphorylated tau levels in the CNS [21,52]. In AD, tau is a key component of neurofibrillary tangles (NFTs) [53]. Recently, a novel concept of a “tauosome” was presented, which refers to the diverse array of tau fragments found in biological fluids. Arastoo et al. identified a collection of high-affinity antibodies that bind to multiple epitopes across the entire tau protein. The antibody panel enables differentiation between these fragments, providing a foundation for the improved diagnosis and monitoring of disease progression [54].

Moreover, several tau isoforms with different numbers of microtubule-binding domains and 29 amino-acid-long inserts exist as tau microtubule-associated proteins (MAPT) [55]. The MAPT gene encodes for tau protein and, in tauopathies, it can be affected by mutations on both the intronic and exonic sequences. In particular, the MAPT mutations can affect the so-called 3R:4R tau ratio, i.e., the ratio of tau with three tubulin-binding repeats (3R) to four tubulin-binding repeats (4R), that is linked to tau aggregation [56,57]. The previous scientific literature reported a detailed list of all the possible protein mutations mentioned above [27,58]. Interestingly, different studies have hypothesized a selective neuronal susceptibility because protein aggregates are frequently detected in particular brain regions associated with specific clinical outcomes, confirming their pathogenic significance in NDDs [59]. 

### 2.2. Functional Imaging: A Tool for Early Dementia Diagnosis

In this context, 2- [Fluorine-18] fluoro-2-deoxy-d-glucose ([18F] FDG) positron emission tomography (PET) represents a functional imaging method for the early or differential diagnosis of dementia [60,61]. In fact, FDG-PET reveals specific regional patterns of cerebral glucose metabolism that may result in the increased or decreased presence of synaptic dysfunction or neural degeneration. AD’s most important FDG PET hallmarks correspond to the hypometabolism observed in the parietotemporal association area, posterior cingulate cortices and precuneus. In mild cognitive impairment (MCI), a syndrome that is diagnosed in individuals who have cognitive deficits that are greater than those that statistically can be expected for their age and education but that do not significantly interfere with their daily activities, the FDG-PET showed a typical hypometabolism pattern in the prodromal phase of AD. Moreover, in the diagnostic field for NDDS, the aggregation protein hallmarks were crucial for obtaining several well-characterized biomarker assays. For AD, PET allows the monitoring of Aβ or tau deposition through specific radiotracers able to link to protein aggregates or receptors. Moreover, the measure of the Aβ42/A40 ratio in cerebrospinal fluid (CSF) represents, together with PET, a useful methodology to detect the onset of Aβ aggregation even decades before clinical disease onset [62]. These approaches play a greater role nowadays in diagnosing and managing AD. In fact, they have been included in several clinical trials aiming at evaluating new molecules for their efficacy in potential therapeutic treatments. These studies are in fact essential to identify biomolecular targets useful for new pharmacological approaches in the field of NDDS. As it concerns the neuronal and synaptic network dysfunction hallmarks, they are linked to specific forms of proteins with differential propensities for aggregation, which occur in particular areas of CNS-causing AD. For example, recent studies highlight the pathogenic relevance of several variables inside and outside the central nervous system, supporting the hypothesis that AD is a syndrome with various etiologies rather than a “heterogeneous” but ultimately unitary disease entity [63,64]. Table 1 reports a summary of the hallmarks and diagnostic strategies for some neurodegenerative diseases.

## 3. Molecular Docking and Neurodrug Development

The increasing prevalence of NDDs poses a considerable challenge to healthcare systems globally, making effective treatment options a pressing necessity. In addressing these challenges, advanced methodologies such as molecular docking play a crucial role in neurodrug development. Molecular docking is a computational technique particularly useful in different fields such as structural molecular biology and drug discovery. Remarkably, the main scope of ligand–protein docking is to predict the key binding mode occurring in a small molecule–biomacromolecule complex using the three-dimensional structure of a known biomacromolecular target (typically a protein or a nucleic acid). As an application of this computer simulation method, docking can be employed to predict the conformation of receptor–ligand complexes with potential biomedical relevance, which is essential in new therapeutics discovery. Importantly, this in silico method has become a predominant part of drug discovery, especially in the first stages of this process when rapidity is particularly needed for the fast screening of libraries of derivatives. Since its initial development more than forty years ago, noteworthy advances in the power of computing hardware and the increasing number and accessibility of small molecule and biomolecular structures have all highly favored the development of optimized molecular docking methods that are routinely employed nowadays by both academia and the industry for which docking has become particularly popular [70]. Over the years, the methodologies by which docking is applied for supporting various drug discovery tasks have changed greatly. In this respect, while molecular docking was originally a stand-alone tool, it is now mostly utilized in conjunction with other computational approaches often included within integrated workflows. Despite its outstanding contribution to drug discovery and development, docking is still far from perfection, with features such as differences in protonation states, active site water molecules and consensus all significantly affecting the results obtainable by this technique [70]. Each molecular docking program takes advantage of one or more specific search algorithms, applied to predict potential conformations of ligand–target binary complexes. A successful docking method generally makes use of a scoring function that effectively searches the high-dimensional space and eventually ranks the possible docking sites. A clear advantage of molecular docking is the possibility of performing screenings of large ligand libraries, ranking the obtained results and generating structural hypotheses about how the compounds bind and possibly inhibit their biological targets, which is of the utmost importance for the optimization of a drug lead. Building the input structure to be used for molecular docking is a step that is as important as docking itself as when it is not correctly performed, it can negatively impact the analysis of the results of probabilistic search techniques [54,71].

### 3.1. Virtual Screening Studies

Virtual screening of ligand databases can be applied to identify new potential inhibitors to be used against targets of pharmaceutical interest. Within this scope, several molecular docking search algorithms use such methods. Optimizing virtual screening and employing relatively fast molecular docking algorithms are both winning strategies that can improve the accuracy of simulation [72]. Additionally, docking can help predict the binding modes and affinities of different nature ligands including small molecules but also peptide structures [73,74] within the binding site of a particular biomolecular receptor set as a target, which often consists of a protein or, more rarely, a DNA [75] structure. Thus, docking results are currently used in drug design to optimize lead compounds in drug discovery. Molecular docking is commonly employed as a standard computational technique in virtual screening studies to search for potentially active molecules or to furnish an interpretation of the binding mode of a bioactive compound within the complex structure it forms with its biomolecular target. As anticipated in the present work, the basic tools used in the molecular docking process include search algorithms and energy evaluation functions that are of pivotal importance in the generation and subsequent evaluation of ligand positions inside the complexes formed by them with a given protein or nucleic target. Features such as the possibility for multiple ligand binding modes, protein flexibility and free energy landscape profiles for binding affinity prediction are crucial and constitute interrelated challenges that a computational researcher needs to address through further methodological developments of molecular docking [76]. As a time-efficient and inexpensive method, molecular docking has been used extensively over the past decades. Nonetheless, several aspects remain challenging, including the ability to easily localize the molecule acting as a ligand within various biomolecular complexes or to locate the correct position of the same molecule within the binding pocket of a particular target. Moreover, it is not always possible to precisely determine the conformation of ligands in an easy and straightforward manner. Nevertheless, new approaches are being continuously developed and presented to the scientific community, aimed at overcoming at least part of the above limits, as testified by the volume of published work on this theme that is proliferating [77]. 

### 3.2. The Application of Molecular Docking in Neurodrug Discovery

**Scheme 1:**
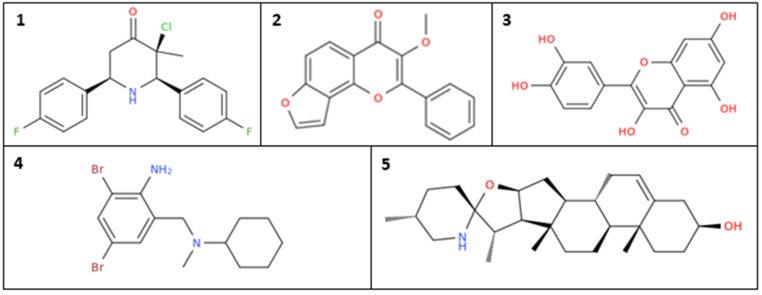
Structural representation of some putative neurodrugs investigated by molecular docking.

Owing to its applicative role, molecular docking has discovered new inhibitors and drugs to treat neurodegenerative diseases, including AD. Since low levels of acetylcholine and abnormal levels of Aβ, tau protein aggregation, inflammation and oxidative stress are all features believed to be associated with AD, research has focused on the cholinergic system and primarily on acetylcholinesterase (AChE) inhibitors [78,79,80]. In general, the AChE inhibitors discovery made significant use of computational modeling and simulation techniques. Among the other techniques, molecular docking made an important contribution not only to the discovery of novel potent acetylcholinesterase inhibitors but also to the elucidation of the mode of binding to AChE, as well as to the identification of the physicochemical requirements for the design of new potential AChE inhibitors [81]. In addition, docking studies were performed to predict and characterize the interactions between new synthetic molecules endowed with potential neuroprotective effects and different proteins involved in AD. Scheme 1 shows some examples of ligands used in molecular dockings. In particular, docking suggested that (2R,3S,6R)-3-chloro-2,6-bis (4-fluorophenyl)-3-methylpiperidin-4-one (**1**, Scheme 1) could have potential in therapy of AD [82]. 

**Figure 1:**
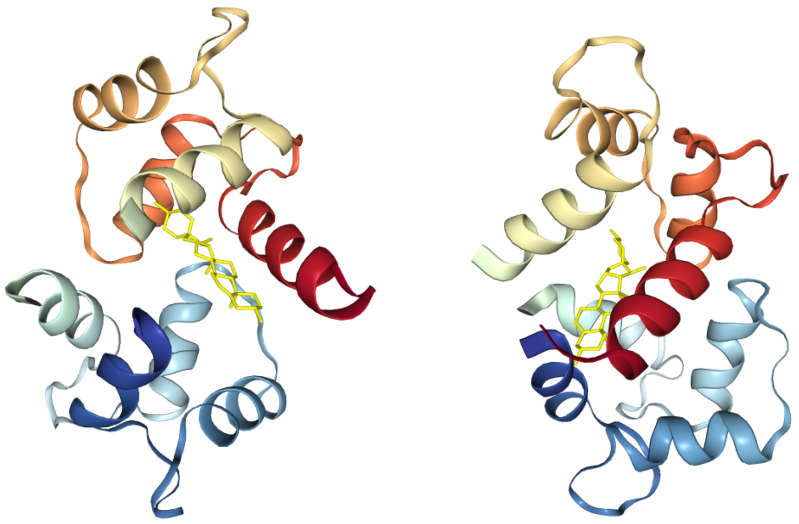
Molecular docking pose views of the complex between solasodine (yellow) and N-type calcium channel (PDB code 3DVE; receptor style: cartoon) as obtained by blind docking with HDOCK server (top-1 pose http://hdock.phys.hust.edu.cn/, accessed on 6 July 2023). The above pictures are property of the authors of the present review article.

Among the drugs usable against AD, but also in the therapy of myasthenia gravis, cholinesterase inhibitors, compounds also known as anticholinesterase and cholinesterase blockers, play a main role as they prevent the breakdown of the neurotransmitters acetylcholine or butyrylcholine [83]. In this respect, the bioactivity scores of several nickel complexes were compared, taking advantage of molecular docking in approaches that tested these metal complexes against human carbonic anhydrase isozyme I, butyrylcholinesterase and AChE, respectively [83]. Currently, available synthetic drugs for treating AD and PD have limited efficacy and are associated with several side effects, whereas natural products remain one of the most important and conservative pharmaceutical sources for treating neurological problems efficiently and with low treatment-associated toxicity [84]. Different flavonoids isolated from several medicinal plants are believed to possess many health benefits. However, their effects on AD and PD are often far from being systematically studied. A large-scale docking study was conducted to evaluate the neuroprotective effects of plant flavonoids, focusing on their molecular targeting against four putative targets in PD and five targets in AD. The results of this in silico study were compared to those of three standard drugs using Molegro Virtual Docker 6.0 and Auto Dock 4.1 software. Docking scores (given as kcal/mol) suggested for one of the plant compounds, namely karanjin (**2**, Scheme 1), revealed relatively higher potencies against the neurodegeneration-related targets than currently used standard drugs. Overall, potential binding affinities from molecular docking and other multiparameter drug efficacy profiles indicated the flavonoid karanjin as an appropriate therapeutic drug for treating AD and PD [84]. A dataset of 142 chalcone derivatives collected from various wild plants was screened by combining a structure-based virtual screening with the ADMET approach, aimed at discovering novel natural chalcones to be used as potential inhibitors of monoamine oxidase-B [85,86], a flavin-dependent enzyme involved in various neurodegenerative diseases. The Gaussian 09.5 software was used to optimize the three-dimensional chemical structures of the compounds using the DFT method, whereas the three-dimensional structure of the enzyme was created using the Modeller V9.23 software. The optimized structures were then subjected to virtual screening with the molecular docking software Autodock Vina 1.2.5 embedded in PyRx. Out of the 142 natural products, 43 were selected based on the binding affinity scores found by docking [87]. Cheminformatics tools and molecular docking simulations were also used to analyze molecules that have been experimentally tested and found to bind α-synuclein [88,89,90]. This led to the identification of six potential natural neuroprotective molecules chemically similar to other known neuroprotective molecules and exhibited high levels of hydrogen bonding with α-synuclein, which suggested them as new effective treatments for PD [91]. Molecular docking studies have also shown that quercetin (**3**, Scheme 1) could be an ideal potential drug targeting aromatic L-amino acid decarboxylases [92] and human catechol *O*-methyltransferases [93], which are putative targets for neuropathologies. Docking made an important contribution to identifying quercetin [94,95,96] as a strong iron chelator with potential in the therapy of PD when administered in the early stages of the disease in combination with L-DOPA [97]. Moreover, a new class of selective antagonists of the *N*-methyl-d-aspartate receptor subunit 2B, correlated with neuropathy, which is related to neurodegenerative diseases, has been developed using molecular modeling. Three-dimensional quantitative structure–activity relationship studies (3D-QSAR) [98] based on comparative molecular field analysis and comparative molecular similarity index analysis models revealed that electrostatic, steric and hydrogen-bond acceptor fields all play important functions. Ultimately, this strategy identified potential drugs with analgesic effects on neuropathic pain. Some of the screened ligands were found to be able to passively cross the blood–brain barrier, with non-toxic effects on the central nervous system, while molecular docking results showed that the active ligands identified from this study as potential analgesic drugs to treat neuropathic pain interacted specifically with the amino acids Gln110, Asp136, Phe176, Glu178, Glu235 and Glu236 of the transport protein [99]. Painful peripheral neuropathy [100,101] was studied through the anti-allodynic and anti-hyperalgesic effects of bromhexine (**4**, Scheme 1) and its pharmacologically active metabolite ambroxol, two repurposed drugs with anti-inflammatory and neuronal sodium channels-blocking activity, that were tested in an oxaliplatin-induced mouse model of neuropathic pain. In this context, molecular docking techniques were employed to predict the molecular targets responsible for the observed in vivo activity of bromhexine [102]. In a study aimed at investigating the neuroprotective effects of an ethanolic extract of *Solanum virginianum* in animal models of chronic structural injury from sciatic nerve-induced neuropathic pain, docking simulations were performed on the three-dimensional structures of voltage-gated N-type, R-type calcium channels and sodium channels. This elucidated the mechanism of action of solasodine (**5**, Scheme 1) [103] showing that 5 is properly located in the FLMM pocket (Phe19, Leu32, Met51 and Met71) of the voltage-gated N-type (Figure 1) and R-type calcium channels, which disabled these calcium channels. Overall, this study confirmed the role of solasodine as a key molecule for the observed neuroprotective effects of eggplant thanks to the information provided by molecular docking [104].

## 4. Machine Learning for Drug Discovery

In addition to molecular docking, ML methods have emerged as a transformative approach in diagnosis and drug discovery, particularly for NDDS. In recent years, ML methods and especially those based on deep learning approaches, have created breakthroughs in several fields, such as speech recognition, image classification and bioinformatics, to name only a few. Moreover, taking advantage of the ever-increasing computing power available in the Graphics Processing Unit (GPU), it was possible to develop a large number of ML methods and applications that enable the processing of large datasets [105,106]. Such techniques and applications have aroused the interest of pharmaceutical companies, which see the great potential for the discovery of new and effective drugs in these emerging technologies. In this context, computational methods, especially ML techniques, can support and improve the therapeutic, diagnostic and disease monitoring process in the case of different neurodegenerative disorders, including AD, PD, Huntington’s disease, amyotrophic lateral sclerosis and multiple system atrophy [107]. 

### 4.1. Drug Design Strategies

In general, a fundamental prerequisite for efficacious drug design consists of the capability to describe or predict the structure of the proteins involved in a biological process. In fact, protein dysfunctions due to structural problems can damage the human body. For this reason, scientists make large use of structural drug design approaches in order to differentiate small molecules in protein targets. Moreover, the prediction of 3D structures is more computationally demanding, especially over de novo predictions. To solve this problem, deep learning and standard feature engineering approaches can be used to predict protein secondary structures [108,109]. Recently, deep learning methods have been applied to improve their performance in predicting three-dimensional protein structures [110]. In this framework, in silico drug discovery exploits multiple biological sources to predict ligand–protein interactions. Nonetheless, a massive corpus of data is needed due to the high number of unknown interactions (19%). Therefore, semi-supervised training techniques [111] have been used to reduce the costs of collecting significant quantities of labelled data. Typically, semi-supervised processes integrate several sources of information, e.g., chemical structures, drug–protein interaction network data and genome sequence data. For example, in [112], the authors achieved good results using drug–protein interactions of various data sets such as ions, enzymes and nuclear receptors. Protein interactions play a crucial role in drug effectiveness. Typically, drug–protein interaction (DPI) data are focused primarily on therapeutic protein targets, whereas the knowledge of non-targets is limited. Computational techniques can fill this lack of knowledge. In [113], the authors used a pool of predictors to estimate the similarity between protein and drug targets. Drug repurposing relies on identifying unexpected drug–protein interactions where the primary drug could be repurposed. However, drug side effects may occur and many human proteins can cause critical consequences. In [114], the authors used a proteomic scale-based method to predict drug–protein interactions. Usually, the results of a drug are assessed by analyzing its biochemical activity. However, accurately matching the therapeutic efficacy of a drug with its biochemical activity is still challenging. Gunther et al. [115] presented a new ML-based technique to predict the therapeutic potential of several psychoactive drugs. To this aim, they used two well-known algorithms (Decision Tree and Random Forest) to analyze microarray data. The experimental results showed that the simpler algorithm (Decision Tree) performed better than an ensemble-based technique like Random Forest. Those results confirmed that ML tools can analyze genomic data to identify the therapeutic efficacy of new drugs.

In drug development, discovering and validating new lead compounds requires an accurate prediction of both the potential drug effectiveness and the related targets. However, due to issues of illness similarity, the prediction accuracy of the treatment effectiveness can be suboptimal, reducing the therapeutic effects of the predicted drugs. Guney et al. [106] defined a drug–disease distance measure employing a network-based method aimed at assessing the interactions occurring between the disease and the drug target. The experimental results (achieved by analyzing data from about 80 diseases treated with more than 230 drugs) demonstrated that the proposed measure led to effective conflict detection and drug repurposing. ML can support the early diagnosis and interpretation of medical images, as well as the discovery and development of new therapies for neurodegenerative diseases. The unifying theme of various ML strategies consists of the integration of multiple high-dimensional data sources, each providing a different view of the pathology and automatically deriving actionable insights [116]. A knowledge graph approach can show non-obvious associations between pathologies and biological drug targets (e.g., new therapeutics based on interactions with proteins known to be mutated in a particular pathological condition). Therefore, identifying protein targets is particularly desirable because, following this information, a single algorithm allows one to predict multiple diseases. ML can also be useful in approaches of large-scale text analysis, as it may suggest proteins related to the disease under examination. In contrast to knowledge graphs, which only consider relationships between entities, this technique uses the entire text as a substrate, leading to a more detailed specification of its biological context [116]. 

### 4.2. Ensuring the Safety of Biomarkers

Biomarkers play a crucial role in drug development, providing vital indicators for assessing a drug’s safety. For this reason, biological and analytical markers must be carefully determined and evaluated. An accurate evaluation of a biomarker requires a rigorous evaluation procedure, which takes into account the unique characteristics of the biomarker, as well as how it is analyzed, integrated and interpreted [117]. Although biomarker safety has been largely investigated, it remains challenging [118]. In this framework, ML-based approaches can provide new and accurate indicators for assessing the security of a drug. In [119], the authors introduced a novel method based on stacked autoencoders to tackle the residue–residue contact prediction problem. The experimental results showed that the features automatically extracted by the autoencoders allowed the authors to perform better than traditional Support Vector Machine (SVM)-based models. Also, protein–protein interactions (PPIs) are crucial in predicting a drug’s safety. In the review presented in [120], the authors summarized state-of-the-art method docking in their analysis of inhibitors in clinical trials against different targets. On the other hand, noting the above considerations and the fact that drug targets are often poorly defined in the literature, [121] Santos et al. presented a comprehensive map of the molecular targets of approved drugs, including about 900 human and pathogen-derived biomolecules. From the derived analysis, they found a continued dominance of privileged target families across disease areas and a growing interest in new first-in-class mechanisms. An innovative ML approach (Variational Autoencoder Modular Bayesian Network) was used to learn a generative model of longitudinal clinical study data, such as those collected from SP513, a double-blinded and placebo-controlled study aimed at comparing two Parkinson’s Disease drugs within an early disease population [122]. Clinical variables collected during the study included baseline demographics, disease duration, UPDRS (Unified Parkinson’s Disease Rating Scale) score [123] and blood biomarkers (such as hemoglobin and creatinine) for safety assessment [122].

### 4.3. Testing Algorithm Effectiveness in Drug Discovery Applications 

**Figure 2:**
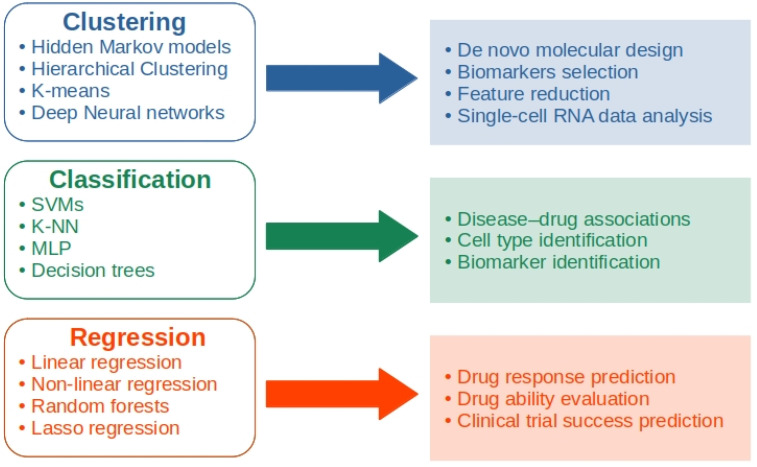
Applications of AI in drug discovery.

In the machine learning field, it has been theoretically proven that no algorithm that outperforms all the current ones exists [124]. Consequently, in any area, for a given task, the development of an effective ML-based tool requires the testing of many algorithms. For this reason, a wide range of algorithms has been used for drug discovery tasks [125]. Figure 2 outlines the applications of AI techniques in the drug discovery field. The following subsections report the results of some popular and widely used ML algorithms.

#### 4.3.1. Support Vector Machine 

A support vector machine (SVM) is a supervised learning algorithm that can be used for problems of binary classification. The SVM algorithm is based on the “support vector” concept, which comprises the data points defining the hyperplanes that linearly separate the feature space areas belonging to the two classes. SVM can also effectively solve non-linear classification problems using kernel functions which implicitly map their inputs into high-dimensional feature spaces [126].This technique has been widely used for drug discovery, exploiting the kernel’s ability to efficaciously recognize data points that are non-linearly separable. In [127], the authors compared Random Forest and SVM for drug discovery from raw data. The authors used those algorithms to predict the radiation protection function and toxicity for radioprotectors targeting the p53 protein. The experimental results demonstrated that SVM outperformed Random Forest in predicting the radiation protection function, whereas the latter was more effective in predicting toxicity. Wang et al. [128] proposed a novel approach to retrieve potential drug target proteins from a protein dataset. The proposed method utilized biochemical attributes to overcome the drawbacks of the techniques employing protein–protein interactive networks. Using a deep learning model, the authors combined a biased SVM with an automatic feature extraction process. The experimental results confirmed the effectiveness of the biochemical attribute-based approach in comparison to other models based on protein–protein interactions. Recently, some authors have analyzed the literature related to the applications of SVM to drug discovery. In [129], the authors review recent applications based on SVM in cancer genomics, leading to new biomarkers, new drug targets and a better understanding of cancer driver genes. In [130], Maltarollo et al. review SVM applications in medicinal chemistry to illustrate their main advantages and disadvantages in drug discovery. From their analysis, the authors found that SVMs are effective in early drug discovery. The Random Forest algorithm is a supervised learning algorithm that builds an ensemble of classifiers combining two well-known strategies for inducing diversity in classifier ensembles: bagging and random subspace [131]. Starting from a dataset consisting of *N* samples, each made of *M* features, the first generated a new dataset by drawing from the dataset *N* samples at random with replacement, whereas the second randomly selected *K* features. Once the models making up the ensemble had been trained, new samples were classified by combining the responses provided by single models trained using the majority rule. Remarkably, RF has been widely used for drug discovery. In [132], the authors present an RF-based approach to improve the selection of molecular descriptors related to ligands of kinases, nuclear hormone receptors and other enzymes. The aim is to reduce computing time by limiting the number of features used for the prediction. 

Rahman et al. [133] faced the problem of designing and training a drug sensitivity predictive model from data from different cancer types. To incorporate heterogeneity in a general purpose ensemble-based approach like RF, they proposed the “Heterogeneity Aware Random Forests” (HARF) method, which computed the weights of the trees making up the ensemble by considering the sample category. The experimental testing on the Cancer Cell Line Encyclopedia (CCLE) and Genomics of Drug Sensitivity in Cancer (GDSC) databases confirmed that HARF outperformed the traditional RF algorithm. In [134], the authors presented a novel technique based on immune network technology to predict the properties of new drugs. They used the RF algorithm to select the most informative descriptors. More recently, RF has also been used for drug sensitivity prediction by simultaneously using classification and regression [135] methods. Indeed, Lehof et al. used the first method to distinguish effective from ineffective drugs, whereas they used regression to quantify the degree of drug effectiveness. Furthermore, to overcome the problems deriving from the high specificity of most anti-cancer drugs, they proposed a novel approach that performed a joint regression and classification analysis. The experimental results confirmed the effectiveness of the proposed method compared to regression or classification alone.

#### 4.3.2. Multilayer Perceptron

A Multilayer Perceptron (MLP) comprises several simple, highly interconnected processing elements called neurons. The output of a neuron is given by the activation function of the weighted sum of its inputs. Typical activation functions are a sigmoid, a hyperbolic tangent and a Rectified Linear Unit (ReLU). MLPs are usually organized in layers. Data samples are presented to the network via the ‘‘input layer’’, whereas the output is provided through an ‘‘output layer’’ that contains several neurons equal to the number of classes to be recognized. On the contrary, the hidden layers perform the non-linear transformation needed to separate the samples from the different classes at hand. Once the network topology has been chosen, an MLP is trained by providing an input set of labelled samples, usually using the back-propagation algorithm [136]. MLP has been widely used for drug design and compound classification. In [137], the authors used an MLP to investigate the psychoactivity level of new cannabinoid compounds. They used molecular descriptors for the psychoactivity of dozens of cannabinoid compounds, which were selected using well-known data analysis techniques. Similarly, de Molfetta et al. [138] used an MLP-based approach to predict the trypanocidal activity of about thirty quinone compounds. To this aim, they trained an MLP to model the nonlinear relationship between the quantum and molecular descriptors and trypanocidal activity. The effectiveness of MLP-based models has also been used to design new drugs against tropical parasitic diseases to reduce the side effects caused by the current medicines, especially during the chronic stages of those diseases [139]. In the last years, Stokes et al. [140] presented a paper on a minimal-input multi-layer perceptron as an approach used to predict drug interactions. This method did not require any structural knowledge of the interacting molecules, only taking advantage of the experimentally accessible physical and chemical properties, which could be experimentally retrieved. Using a large dataset (comprising more than 600,000 entries) containing drug interactions and their related properties, the same authors achieved an accuracy of 0.968 on unseen samples of interactions between drugs on which the model was trained, whereas an accuracy of 0.942 on the unseen samples of interactions was found between unseen drugs. Those promising results confirmed the great potential of the proposed approach for high generalized predictive accuracy.

#### 4.3.3. Deep Learning

Deep learning is a subfield of ML that uses the training of artificial neural networks in order to solve complex tasks such as speech and image recognition, decision-making and natural language processing, among others. The adjective “deep” refers to the multiple layers of interconnected nodes that form these neural networks, making them capable of learning and representing highly complex patterns and relationships in data. Thanks to the explosion of large data and the ever-growing advancements achieved in computing power, deep learning has become a powerful technique to be used for solving different problems in various industries, from finance and healthcare to gaming and entertainment [141]. In [142], the authors proposed a two-step strategy to predict protein secondary structures using an MLP approach. In the first step, the training data were employed to learn the parameters of models based on the clonal selection algorithm (abbreviated as CSA), which is based on the natural metaphors of live immune systems. On the other hand, the second step is used as input data provided by CSA given to an MLP classifier. The experimental results confirmed the effectiveness of the CSA-based preprocessing. Gómez-Bombarelli et al. [143] presented a new method to convert discrete representations of molecules to and from a multidimensional continuous model. The proposed approach trained a deep neural network on a large dataset to efficaciously explore the non-linear and complex search space of molecules. The experimental results seemed to confirm the effectiveness of the proposed approach in discovering optimized drug candidates. 

The deep learning model training requirement of a large amount of data contrasts with the current situation of drug discovery pipelines, in which only tiny and often biased data are available. This problem can be solved by adopting the federated learning approach, a new training strategy based on distributed data that allows users to preserve sensitive information. This emerging decentralized approach is expected to improve the performance of ML-based drug discovery dramatically. In [144], the authors simulated several scenarios of federated learning and found that federated training has a better regularization effect than centralized training, especially when the pooled datasets are highly biased. The recently introduced generative AI technology has also been affecting the fields of drug discovery and design by making the search process more efficient. In [145], the authors present a systematic review of generative AI for drug discovery. From the study, the authors found that six algorithms are widely used, including long short-term memory recurrent neural networks (LSTM-RNNs), variational autoencoders (VAEs), generative adversarial networks (GANs) and adversarial autoencoders (AAEs). The authors also identified the main challenges in the field. These challenges include the homogeneity of generated molecular libraries, deficient synthesizability, model interpretability and the incapacity for multi-property optimization and uncertainty in model evaluation. 

Novel drug discovery in the neurodegenerative disease area has yet to receive much attention from drug discovery researchers. Since central nervous system therapeutics are challenging to generate due to the presence of obstacles such as the blood–brain barrier (BBB) and P-glycoprotein, an efflux transporter, which first makes drugs as substrate and then effluxes them out, the development of new deep learning-based algorithms and the use of established methods play essential roles in the generation of new chemical entities to be used in the neurodegeneration therapeutic area [146].

### 4.4. Machine Learning for Neurodrug Discovery

More than fifty years ago, quantitative structure–activity relationship (QSAR) analysis was presented as the first method that was able to aid ligand-based drug development. Leveraging mathematical models, QSAR was built up to predict the biological behavior of novel lead compounds. This technique is widespread in pharmaceutical industries and academic institutes thanks to its time-, resource-, cost- and labor-saving characteristics in searching for novel drugs [147,148]. Initially, QSAR modeling was based on simple regression techniques and its classification was performed on a limited series of similar compounds. The aim was to identify a statistically meaningful association between the chemical structure and continuous variable (pIC50, Ki, pEC50, etc.) or categorical/binary (inactive, active, nontoxic, toxic, etc.) biological properties [149]. Today, several virtual screening (VS) techniques make use of the QSAR methodology with a significant evolution over time. The expansion, development and diversification of QSAR modeling led to the VS of vast data sets composed of thousands of different molecules. All this took place due to the explosion of high-throughput screening technology and several ML approaches [149,150,151,152]. Currently, the initial step necessary in developing a QSAR model is gathering pertinent chemogenomic data available from the literature and various databases. Finally, taking advantage of ML approaches, chemical descriptors are computed based on various representations of undefinable sets of molecular structures and then connected with the corresponding biological profile. Several molecules have been identified as potential candidates for treating neurological disorders using artificial intelligence. Here, we report the case of Elenbecestat and DSP-1181. Developed by Sumitomo Dainippon Pharma in collaboration with the AI drug discovery company Exscientia, DSP-1181 is an AI-designed molecule for treating obsessive-compulsive disorder [153]. It became the first AI-designed drug to enter human clinical trials in 2019 [154]. DSP-1181 was developed using an AI-based drug discovery platform called Centaur Chemist™, designed by Exscientia. The platform utilizes ML algorithms to analyze large amounts of data and predict new lead compound properties and therapeutic potential. In the case of DSP-1181, the AI algorithm was trained on a vast range of data, including safety and pharmacological data, to generate novel structures with desirable properties for application in the therapy for obsessive-compulsive disorder (OCD). The AI-driven system then narrowed the potential candidates to a molecule considered suitable for further development. Therefore, Sumitomo Dainippon Pharma performed preclinical studies aimed at evaluating the efficacy and safety of DSP-1181 before proceeding to conclusive human clinical trials. The drug development and entry into clinical trials marked a significant milestone in the application of AI in the neurodrug discovery and development processes. Although DSP-1181 showed promise in preclinical and early clinical trials, further research and larger-scale trials are in urgent need to determine its safety, as well as to confirm its efficacy and potential as a treatment for OCD or other related conditions. Another example was represented by the molecule known by the name of Elenbecestat. Elenbecestat is an inhibitor of the enzyme oral beta-secretase 1 (BACE1) that was discovered using ML algorithms [155]. Famously, BACE1 plays a fundamental role in the production of Aβ, which, as already explained in a previous section of this review, is thought to contribute to AD. Elenbecestat is currently undergoing assessment in phase III clinical trials. It is important to note that those presented herein are just a few examples of the drugs discovered using ML methodologies to treat neuropathies. The use of ML and other in silico approaches is an attractive area of scientific research that is very active in drug discovery and holds great promise for developing new therapeutics for neurodegenerative disorders [156]. It is worth noting that while AI can accelerate the drug discovery process, further research and clinical trials are also needed to evaluate their safety and efficacy in humans through computational drug discovery approaches. 

## 5. Impact of State-of-the-Art Computational Approaches in Neurodrug Discovery

The omics field (including genomics, proteomics and transcriptomics) is experiencing a surge in data generation due to advancements in high-throughput sequencing technologies. This surge of data offers valuable opportunities for predictive modeling in precision medicine, especially for understanding complex diseases like Alzheimer’s disease. Yet, it also poses considerable challenges when it comes to analyzing and interpreting the extensive datasets generated. Traditional ML approaches have had some success in developing predictive models for omics data but often struggle to fully capture the intricate relationships within the datasets, leading to poorly accurate predictions.

Recent advancements in deep learning, particularly through the use of convolutional neural networks (CNNs), are revolutionizing predictive modeling in this area. Innovative transformation methods, such as DeepInsight, allow for the conversion of omics data, which are typically structured in tabular formats into image-like representations [157]. This transformation enables CNNs to effectively identify latent features within the data, enhancing predictive accuracy while also benefiting from transfer learning. This results in reduced computational time and improved overall performance. Despite these advancements, the integration of CNNs into predictive analyses of omics data poses several challenges, including concerns regarding model interpretability, data heterogeneity, and the immense volume of information involved. Addressing these issues necessitates a collaborative approach that brings together experts in ML, bioinformatics, biology and medicine. Notably, the work by Panizza et al. highlights how deep learning frameworks can identify proteomic drivers of AD, further emphasizing the potential of these innovative computational methods to uncover novel drug targets and enhance our understanding of the disease mechanisms involved in AD [158]. In summary, as efforts intensify to generate and analyze omics data related to AD, the integration of advanced computational techniques holds promise for identifying new therapeutic avenues and improving predictive modeling in this critical area of research.

ML techniques can analyze omics data, clinical trial data and even imaging data to identify potential therapeutic targets and predict drug responses [159,160,161]. Notwithstanding the significant promise of machine learning in drug discovery for neurodegenerative diseases, to the best of our knowledge, no drugs identified through these techniques have yet reached advanced stages of clinical trials. This gap between potential and realization can stem from a variety of factors that hinder the translation of ML insights into viable therapeutics. Firstly, the quality and availability of data necessary for training ML models are often inadequate, as high-quality biological data from human tissues are limited. Secondly, NDDS are complex, involving multiple genetic and environmental factors, which complicates the ability of ML models to accurately identify therapeutic targets and compounds. Additionally, there is a notable translational gap between preclinical findings and clinical outcomes; many compounds that appear promising in vitro or in animal models fail to translate effectively to human trials due to physiological differences. Moreover, integrating ML with other methodologies, such as systems biology and structural biology, is essential for understanding drug interactions comprehensively, but takes time and collaborative effort. Overcoming these obstacles is crucial for realizing the full potential of ML in developing new therapies for NDDS. Although there are currently no advanced-stage clinical compounds for NDDS identified through these methodologies, numerous studies in the literature demonstrate truly promising data. These findings highlight the potential of ML to uncover new therapeutic targets and candidates, paving the way for future drug development efforts in this challenging field.

For example, Bagget and Nath combined computational docking and machine learning-based screening of compound libraries in order to identify active small molecule ligands of “disordered” proteins [162]. Using this protocol on an amyloid-forming segment of the microtubule-associated protein tau, they successfully discovered new, chemically diverse tau ligands. Among these, they identified an inhibitor that slows down the aggregation process in vitro.

### 5.1. Drug Repurposing in AD

Another highly promising opportunity in drug discovery offered by ML methodologies is drug repurposing, i.e., the use of an existing drug in a new way, perhaps at a different dose or in a different formulation [163]. Alternatively, repurposing can be employed to evaluate a therapeutic concept, which can then be further developed through additional medicinal chemistry and functional testing to become a new molecular entity. For example, very recently, Rodriguez et al. developed DRIAD, a ML framework designed to assess potential links between a disease and any biological process defined by a set of genes. DRIAD was used to identify associations between the pathological stages of AD and genes that show altered expression when a small molecule drug is introduced to a culture of terminally differentiated neuronal progenitor cells, consisting of a mixture of neurons, astrocytes and oligodendrocytes [164]. They screened a total of 88 molecules and found that 33 of them were FDA-approved drugs that can potentially be repurposed directly in the treatment of AD. These methodologies are very attractive because repurposing drugs that have already been approved by the Food and Drug Administration (FDA) for a different indication is more cost-effective, comes with known potential toxicities and can achieve a higher success rate (30%) compared to the development of a new molecular entity. Similarly, Oliveros et al. implemented an innovative multi-scale predictive modeling approach that combines ML with biophysical methods and systems pharmacology to evaluate potential drugs for AD [165]. This model highlighted ibudilast as a promising candidate for repurposing in the treatment of AD. Ibudilast acts as a multi-target drug, functioning both as a phosphodiesterase inhibitor and a toll-like receptor 4 (TLR4) antagonist [166]. Additionally, the predictions suggested that ibudilast may inhibit off-target kinases, such as IRAK1 and GSG2 [165]. In Japan and other Asian countries, ibudilast is already approved for the treatment of asthma and stroke, thanks to its anti-inflammatory properties [167].

### 5.2. Target Discovery in AD

Target discovery is a critical aspect of the drug discovery process, particularly for neurodegenerative diseases like AD. It involves identifying and validating biological targets that can be modulated to achieve therapeutic effects. Effective target discovery lays the groundwork for the development of both symptomatic and disease-modifying drugs. Symptomatic drugs, like cholinesterase inhibitors (e.g., donepezil) and memantine, have been the cornerstone of AD treatment for years, focusing on alleviating symptoms without altering the underlying disease progression [168,169]. In contrast, disease-modifying drugs aim to intervene in the pathophysiological mechanisms of AD, potentially slowing or halting disease progression [170,171]. 

Historically, disease-modifying therapies have been elusive, with many clinical trials failing to demonstrate efficacy in altering the course of AD. However, the recent and controversial approvals of lecanemab and donanemab by the FDA in the USA in January 2023 and July 2024, respectively, have marked a significant shift in the treatment landscape [172,173,174,175]. Both drugs are monoclonal antibodies that target Aβ. Lecanemab and donanemab aim to reduce the build-up of Aβ plaques, which are thought to contribute to the neurodegenerative processes underlying cognitive decline in AD [176].

Lecanemab, co-developed by Eisai and Biogen, has been shown in clinical trials to slow cognitive decline in patients with early-stage AD by binding to soluble forms of Aβ and facilitating the clearance of Aβ aggregates [177]. In the pivotal CLARITY AD trial, lecanemab was reported to reduce the rate of cognitive decline by approximately 27% compared to a placebo used over the course of 18 months [178]. Despite these promising results, the drug has sparked debate in the scientific community due to its side effects, such as amyloid-related imaging abnormalities (ARIA), which can manifest in brain swelling or microhemorrhages [179]. The need for careful patient monitoring and potential adverse effects have raised questions about the risk–benefit ratio for patients, especially those with certain genetic risk factors like the APOE4 allele, NBK605938.

Similarly, donanemab, developed by Eli Lilly, targets a specific form of Aβ called N3pG, which is thought to be an especially pathogenic species of the amyloid plaques in AD [172]. The TRAILBLAZER-ALZ trials have demonstrated that donanemab can reduce amyloid burden in the brain and slow clinical progression in patients with early symptomatic AD [180]. The drug’s efficacy in slowing disease progression has been highlighted as a significant advancement, with reported reductions in the clinical decline of 35% in patients with low-to-intermediate tau pathology [181]. However, donanemab has also been associated with ARIA and other side effects, leading to similar concerns about the potential risks of treatment [182]. The approval of these drugs has been met with both hope and skepticism [174]. On one hand, they represent a long-awaited step forward in targeting the underlying mechanisms of AD, offering potential hope for patients beyond symptomatic relief. On the other hand, critics have pointed out that the degree of cognitive improvement observed in trials may not translate into substantial clinical benefit for all patients, especially given the serious side effects. Additionally, the high cost of these treatments, alongside the need for regular brain imaging to monitor for ARIA, poses challenges for widespread accessibility and adoption in clinical practice. The approvals have also sparked discussions about the criteria used by regulatory agencies like the FDA and whether the emphasis should be on clinical efficacy or the ability to clear amyloid plaques as a surrogate marker. While the long-term impact of lecanemab and donanemab remains to be seen, their introduction has underscored the importance of continued efforts in target discovery and drug development. As the field evolves, the combination of traditional and computational methods in drug discovery will be essential for refining these approaches and ensuring that future therapies are both safe and effective.

## 6. Conclusions

In conclusion, computational methods are transforming drug discovery not only for neurodegenerative diseases but also across various therapeutic areas, presenting new opportunities for identifying therapeutic targets and creating effective treatments. Herein, we summarized some of the most innovative computational-based strategies to develop neuropharmaceuticals. In particular, we discussed the current application of molecular docking and artificial intelligence for ligand- and target-based approaches in novel drug development, highlighting the critical role of in silico methods in neurodrug discovery. In recent years, the availability of relevant data and effective docking softwares and ML-based algorithms has greatly improved our comprehensive understanding of advanced neurodegenerative mechanisms. These trending applications are now gaining popularity, fueled by the unprecedented success of artificial intelligence and ML-based approaches. The future of ML in neurodegenerative drug discovery likely lies in its integration with other technologies, such as advanced imaging and personalized medicine, enabling more precise targeting of disease mechanisms. While the journey is far from complete, the potential for ML to contribute to breakthroughs in treating neurodegenerative diseases continues to inspire optimism within the scientific community. AI, ML, and molecular docking are intrinsically connected in modern neurodrug development. In our opinion, the synergy between these technologies enhances the drug discovery process by optimizing docking protocols and accurately predicting ligand binding affinities. This integrated approach allows for more efficient virtual screening of compounds, informed decision-making, and identification of potential biomarkers. Additionally, AI and ML can predict possible side effects based on molecular structures, ultimately improving patient safety. Together, we believe these advancements will significantly accelerate the discovery of effective treatments for neurological disorders. As we venture into this new era of drug discovery, the fusion of molecular docking and artificial intelligence with innovative experimental techniques may not only revolutionize our understanding of these complex diseases but also unveil unexpected therapeutic pathways that could redefine treatment paradigms. Therefore, ongoing developments in this area will shortly enable the scientific community to develop more specific and potent modulators to be directed against multiple proteins involved in CNS diseases.

## Figures and Tables

**Table 1:** Summary giving information about the hallmarks, neuropathology and current diagnostic markers for Alzheimer’s, Parkinson’s, amyotrophic lateral sclerosis, Huntington’s disease and multiple sclerosis. Please note that this table intends to provide a simplified overview, whereas each neurodegenerative disease involves complex mechanisms and additional factors.

Neurodegenerative Disease	Hallmarks and Neuropathology	Current Diagnostic Markers
Alzheimer’s Disease	1. Amyloid plaques: extracellular deposits of beta-amyloid protein. 2. Neurofibrillary tangles: intracellular twisted fibres of tau protein. 3. Neuronal loss: progressive degeneration and death of brain cells. 4. Inflammation: activation of immune cells in the brain. 5. Cholinergic dysfunction: impairment of acetylcholine-producing neurons.	1. Amyloid beta (Aβ) protein: increased levels in cerebrospinal fluid (CSF) and detection via positron emission tomography (PET) scans. 2. Tau protein: elevated levels in CSF and detection via PET scans. 3. Apolipoprotein E (APOE) genotype: Presence of APOE ε4 allele increases risk [65].
Parkinson’s Disease	1. Lewy bodies: abnormal protein aggregates composed of α-synuclein. 2. Dopaminergic cell loss: degeneration of dopamine-producing neurons. 3. Motor symptoms: tremors, rigidity, bradykinesia and postural instability. 4. Neuroinflammation: activation of immune response in the brain.	1. Dopamine transporter (DAT) imaging: reduced uptake of radiolabeled tracer in the striatum on single-photon emission computed tomography (SPECT) or PET scans. 2. α-synuclein (Lewy bodies): detection of abnormal α-synuclein aggregates in CSF or through imaging techniques. 3. Genetic testing: identification of specific mutations, such as in the LRRK2 or PARKIN genes [66].
Huntington’s Disease	1. CAG repeat expansion: abnormal repetition of the CAG nucleotide sequence in the huntingtin gene. 2. Protein aggregation: accumulation of mutant huntingtin protein in neurons. 3. Basal ganglia degeneration: selective atrophy of brain structures involved in movement control. 4. Motor and cognitive impairments: jerky movements, cognitive decline, psychiatric symptoms.	1. Genetic testing: detection of the CAG repeat expansion in the Huntington gene [67]. 2. Clinical presentation: motor, cognitive and psychiatric symptom assessment.
Amyotrophic Lateral Sclerosis (ALS)	1. Motor neuron degeneration: progressive loss of upper and lower motor neurons. 2. Muscle weakness and atrophy: difficulty with voluntary muscle control. 3. Glutamate excitotoxicity: excessive release of glutamate, causing neuronal damage. 4. Neuroinflammation: activation of immune response in the central nervous system.	1. Electromyography (EMG): assessment of electrical activity in muscles. 2. Neuroimaging: Magnetic Resonance Imaging (MRI) scans to rule out other conditions. 3. Genetic testing: identification of gene mutations such as SOD1, C9orf72 and others [68].
Multiple Sclerosis	1. Demyelination: damage to the protective myelin sheath around nerve fibres. 2. Immune system dysfunction: attack by the immune system on the central nervous system. 3. Inflammation and scarring: scar tissue formation (sclerosis) in affected areas. 4. Neurological deficits: varying symptoms depending on the affected nerve fibers.	1. MRI detection of demyelination, presence of lesions and distribution in the central nervous system. 2. CSF analysis: increased levels of immunoglobulin G (IgG) and oligoclonal bands. 3. Evoked potentials: measurement of electrical activity in response to sensory stimulation [69]

## References

[1] Dehhaghi M., Mohammadipanah F., Guillemin G.J. (2018). Myxobacterial natural products: An under-valued source of products for drug discovery for neurological disorders. NeuroToxicology.

[2] Gomes N.G.M., Campos M.G., Órfão J.M.C., Ribeiro C.A.F. (2009). Plants with neurobiological activity as potential targets for drug discovery. Prog. Neuro-Psychopharmacol. Biol. Psychiatry.

[3] Marasco D., Vicidomini C., Krupa P., Cioffi F., Huy P.D.Q., Li M.S., Florio D., Broersen K., De Pandis M.F., Roviello G.N. (2021). Plant isoquinoline alkaloids as potential neurodrugs: A comparative study of the effects of benzo[c]phenanthridine and berberine-based compounds on β-amyloid aggregation. Chem.-Biol. Interact..

[4] Rezaul Islam M., Akash S., Murshedul Islam M., Sarkar N., Kumer A., Chakraborty S., Dhama K., Ahmed Al-Shaeri M., Anwar Y., Wilairatana P. (2024). Alkaloids as drug leads in Alzheimer’s treatment: Mechanistic and therapeutic insights. Brain Res..

[5] Kanakala G.C., Devata S., Chatterjee P., Priyakumar U.D. (2024). Generative artificial intelligence for small molecule drug design. Curr. Opin. Biotechnol..

[6] Srivastava P., Tiwari A. (2017). Critical Role of Computer Simulations in Drug Discovery and Development. Curr. Top. Med. Chem..

[7] Raees M., Meijerink I., Lykourentzou I., Khan V.-J., Papangelis K. (2024). From explainable to interactive AI: A literature review on current trends in human-AI interaction. Int. J. Hum.-Comput. Stud..

[8] Pinzi L., Rastelli G. (2019). Molecular Docking: Shifting Paradigms in Drug Discovery. Int. J. Mol. Sci..

[9] Van Schependom J., D’haeseleer M. (2023). Advances in Neurodegenerative Diseases. J. Clin. Med..

[10] Livingston G., Sommerlad A., Orgeta V., Costafreda S.G., Huntley J., Ames D., Ballard C., Banerjee S., Burns A., Cohen-Mansfield J. (2017). Dementia prevention, intervention, and care. Lancet.

[11] Knopman D.S., Amieva H., Petersen R.C., Chételat G., Holtzman D.M., Hyman B.T., Nixon R.A., Jones D.T. (2021). Alzheimer disease. Nat. Rev. Dis. Primers.

[12] Scheltens P., De Strooper B., Kivipelto M., Holstege H., Chételat G., Teunissen C.E., Cummings J., van der Flier W.M. (2021). Alzheimer’s disease. Lancet.

[13] Poewe W., Seppi K., Tanner C.M., Halliday G.M., Brundin P., Volkmann J., Schrag A.-E., Lang A.E. (2017). Parkinson disease. Nat. Rev. Dis. Primers.

[14] Przedborski S. (2017). The two-century journey of Parkinson disease research. Nat. Rev. Neurosci..

[15] Long J.M., Holtzman D.M. (2019). Alzheimer Disease: An Update on Pathobiology and Treatment Strategies. Cell.

[16] Fang E.F., Lautrup S., Hou Y., Demarest T.G., Croteau D.L., Mattson M.P., Bohr V.A. (2017). NAD+ in Aging: Molecular Mechanisms and Translational Implications. Trends Mol. Med..

[17] Walker K.A., Chen J., Shi L., Yang Y., Fornage M., Zhou L., Schlosser P., Surapaneni A., Grams M.E., Duggan M.R. (2023). Proteomics analysis of plasma from middle-aged adults identifies protein markers of dementia risk in later life. Sci. Transl. Med..

[18] Kamboh M.I., Minster R.L., Kenney M., Ozturk A., Desai P.P., Kammerer C.M., DeKosky S.T. (2006). Alpha-1-antichymotrypsin (ACT or SERPINA3) polymorphism may affect age-at-onset and disease duration of Alzheimer’s disease. Neurobiol. Aging.

[19] Bellenguez C., Küçükali F., Jansen I.E., Kleineidam L., Moreno-Grau S., Amin N., Naj A.C., Campos-Martin R., Grenier-Boley B., Andrade V. (2022). New insights into the genetic etiology of Alzheimer’s disease and related dementias. Nat. Genet..

[20] Kamboh M.I., Demirci F.Y., Wang X., Minster R.L., Carrasquillo M.M., Pankratz V.S., Younkin S.G., Saykin A.J., Jun G., Alzheimer’s Disease Neuroimaging Initiative (2012). Genome-wide association study of Alzheimer’s disease. Transl. Psychiatry.

[21] Silva M.V.F., Loures C.d.M.G., Alves L.C.V., de Souza L.C., Borges K.B.G., Carvalho M.d.G. (2019). Alzheimer’s disease: Risk factors and potentially protective measures. J. Biomed. Sci..

[22] Breijyeh Z., Karaman R. (2020). Comprehensive Review on Alzheimer’s Disease: Causes and Treatment. Molecules.

[23] Iravanpour F., Dargahi L., Rezaei M., Haghani M., Heidari R., Valian N., Ahmadiani A. (2021). Intranasal insulin improves mitochondrial function and attenuates motor deficits in a rat 6-OHDA model of Parkinson’s disease. CNS Neurosci. Ther..

[24] Caberlotto L., Nguyen T.P., Lauria M., Priami C., Rimondini R., Maioli S., Cedazo-Minguez A., Sita G., Morroni F., Corsi M. (2019). Cross-disease analysis of Alzheimer’s disease and type-2 diabetes highlights the role of autophagy in the pathophysiology of two highly comorbid diseases. Sci. Rep..

[25] Baduini I.R., Castro Vildosola J.E., Kavehmoghaddam S., Kiliç F., Nadeem S.A., Nizama J.J., Rowand M.A., Annapureddy D., Bryan C.A., Do L.H. (2024). Type 2 diabetes mellitus and neurodegenerative disorders: The mitochondrial connection. Pharmacol. Res..

[26] Sensi S.L., Russo M., Tiraboschi P. (2023). Biomarkers of diagnosis, prognosis, pathogenesis, response to therapy: Convergence or divergence? Lessons from Alzheimer’s disease and synucleinopathies. Precision Medicine in Neurodegenerative Disorders, Part I.

[27] Tsoi P.S., Quan M.D., Ferreon J.C., Ferreon A.C.M. (2023). Aggregation of Disordered Proteins Associated with Neurodegeneration. Int. J. Mol. Sci..

[28] Zhang A., Pan C., Wu M., Lin Y., Chen J., Zhong N., Zhang R., Pu L., Han L., Pan H. (2024). Causal association between plasma metabolites and neurodegenerative diseases. Prog. Neuro-Psychopharmacol. Biol. Psychiatry.

[29] Cheslow L., Snook A.E., Waldman S.A. (2024). Biomarkers for Managing Neurodegenerative Diseases. Biomolecules.

[30] Fornito A., Zalesky A., Breakspear M. (2015). The connectomics of brain disorders. Nat. Rev. Neurosci..

[31] Starr A., Sattler R. (2018). Synaptic dysfunction and altered excitability in C9ORF72 ALS/FTD. Brain Res..

[32] Pohl C., Dikic I. (2019). Cellular quality control by the ubiquitin-proteasome system and autophagy. Science.

[33] Komatsu M., Waguri S., Chiba T., Murata S., Iwata J.-i., Tanida I., Ueno T., Koike M., Uchiyama Y., Kominami E. (2006). Loss of autophagy in the central nervous system causes neurodegeneration in mice. Nature.

[34] Ghosh A., Singh S. (2022). Regulation of Microtubule: Current Concepts and Relevance to Neurodegenerative Diseases. CNS Neurol. Disord.-Drug Targets.

[35] Cartelli D., Cappelletti G. (2016). Microtubule Destabilization Paves the Way to Parkinson’s Disease. Mol. Neurobiol..

[36] McDonald T.S., Lerskiatiphanich T., Woodruff T.M., McCombe P.A., Lee J.D. (2022). Potential mechanisms to modify impaired glucose metabolism in neurodegenerative disorders. J. Cereb. Blood Flow Metab..

[37] Procaccini C., Santopaolo M., Faicchia D., Colamatteo A., Formisano L., de Candia P., Galgani M., De Rosa V., Matarese G. (2016). Role of metabolism in neurodegenerative disorders. Metabolism.

[38] Tiwari V., Wilson D.M. (2019). DNA Damage and Associated DNA Repair Defects in Disease and Premature Aging. Am. J. Hum. Genet..

[39] Swinnen B., Robberecht W., Van Den Bosch L. (2019). RNA toxicity in non-coding repeat expansion disorders. EMBO J..

[40] Sun Y., Yu H., Guan Y. (2023). Glia Connect Inflammation and Neurodegeneration in Multiple Sclerosis. Neurosci. Bull..

[41] Anwar S., Rivest S. (2020). Alzheimer’s disease: Microglia targets and their modulation to promote amyloid phagocytosis and mitigate neuroinflammation. Expert Opin. Ther. Targets.

[42] Wilson D.M., Cookson M.R., Van Den Bosch L., Zetterberg H., Holtzman D.M., Dewachter I. (2023). Hallmarks of neurodegenerative diseases. Cell.

[43] Taylor J.P., Hardy J., Fischbeck K.H. (2002). Toxic Proteins in Neurodegenerative Disease. Science.

[44] Cras P., van Harskamp F., Hendriks L., Ceuterick C., van Duijn C.M., Stefanko S.Z., Hofman A., Kros J.M., Van Broeckhoven C., Martin J.J. (1998). Presenile Alzheimer dementia characterized by amyloid angiopathy and large amyloid core type senile plaques in the APP 692Ala→Gly mutation. Acta Neuropathol..

[45] Pascoal T.A., Aguzzoli C.S., Lussier F.Z., Crivelli L., Suemoto C.K., Fortea J., Rosa-Neto P., Zimmer E.R., Ferreira P.C.L., Bellaver B. (2024). Insights into the use of biomarkers in clinical trials in Alzheimer’s disease. EBioMedicine.

[46] Thomas J., Wilson S. (2024). Molecular and therapeutic targets for amyloid-beta plaques in Alzheimer’s disease: A review study. Basic Clin. Neurosci..

[47] Selkoe D.J. (2001). Alzheimer’s Disease: Genes, Proteins, and Therapy. Physiol. Rev..

[48] Nalivaeva N.N., Turner A.J. (2013). The amyloid precursor protein: A biochemical enigma in brain development, function and disease. FEBS Lett..

[49] Belyaev N.D., Kellett K.A.B., Beckett C., Makova N.Z., Revett T.J., Nalivaeva N.N., Hooper N.M., Turner A.J. (2010). The Transcriptionally Active Amyloid Precursor Protein (APP) Intracellular Domain Is Preferentially Produced from the 695 Isoform of APP in a β-Secretase-dependent Pathway. J. Biol. Chem..

[50] Zhang X., Zhang C.M., Prokopenko D., Liang Y., Zhen S.Y., Weigle I.Q., Han W., Aryal M., Tanzi R.E., Sisodia S.S. (2021). An APP ectodomain mutation outside of the Aβ domain promotes Aβ production in vitro and deposition in vivo. J. Exp. Med..

[51] Cruts M., Theuns J., Van Broeckhoven C. (2012). Locus-specific mutation databases for neurodegenerative brain diseases. Hum. Mutat..

[52] Muratore C.R., Rice H.C., Srikanth P., Callahan D.G., Shin T., Benjamin L.N.P., Walsh D.M., Selkoe D.J., Young-Pearse T.L. (2014). The familial Alzheimer’s disease APPV717I mutation alters APP processing and Tau expression in iPSC-derived neurons. Hum. Mol. Genet..

[53] Goedert M., Spillantini M.G., Jakes R., Rutherford D., Crowther R.A. (1989). Multiple isoforms of human microtubule-associated protein tau: Sequences and localization in neurofibrillary tangles of Alzheimer’s disease. Neuron.

[54] Arastoo M., Penny L.K., Lofthouse R., Abdallah A., Abrahamsson A., Marini P., Melis V., Riedel G., Harrington C.R., Wischik C.M. (2024). High-affinity antibodies specific to the core region of the tau protein exhibit diagnostic and therapeutic potential for Alzheimer’s disease. Alzheimers Res. Ther..

[55] Ballatore C., Lee V.M.Y., Trojanowski J.Q. (2007). Tau-mediated neurodegeneration in Alzheimer’s disease and related disorders. Nat. Rev. Neurosci..

[56] Sergeant N., David J.P., Lefranc D., Vermersch P., Wattez A., Delacourte A. (1997). Different distribution of phosphorylated tau protein isoforms in Alzheimer’s and Pick’s diseases. FEBS Lett..

[57] Stanford P.M., Halliday G.M., Brooks W.S., Kwok J.B.J., Storey C.E., Creasey H., Morris J.G.L., Fulham M.J., Schofield P.R. (2000). Progressive supranuclear palsy pathology caused by a novel silent mutation in exon 10 of the tau gene. Brain.

[58] Andrade-Guerrero J., Santiago-Balmaseda A., Jeronimo-Aguilar P., Vargas-Rodríguez I., Cadena-Suárez A.R., Sánchez-Garibay C., Pozo-Molina G., Méndez-Catalá C.F., Cardenas-Aguayo M.-d.-C., Diaz-Cintra S. (2023). Alzheimer’s Disease: An Updated Overview of Its Genetics. Int. J. Mol. Sci..

[59] Dugger B.N., Dickson D.W. (2017). Pathology of Neurodegenerative Diseases. Cold Spring Harb. Perspect. Biol..

[60] Phelps M.E., Huang S.C., Hoffman E.J., Selin C., Sokoloff L., Kuhl D.E. (1979). Tomographic measurement of local cerebral glucose metabolic rate in humans with (F-18)2-fluoro-2-deoxy-D-glucose: Validation of method. Ann. Neurol..

[61] Kato T., Inui Y., Nakamura A., Ito K. (2016). Brain fluorodeoxyglucose (FDG) PET in dementia. Ageing Res. Rev..

[62] Jansen W.J., Janssen O., Tijms B.M., Vos S.J.B., Ossenkoppele R., Visser P.J., Aarsland D., Alcolea D., Altomare D., von Arnim C. (2022). Prevalence Estimates of Amyloid Abnormality Across the Alzheimer Disease Clinical Spectrum. JAMA Neurol..

[63] Singh M.K., Shin Y., Ju S., Han S., Kim S.S., Kang I. (2024). Comprehensive Overview of Alzheimer’s Disease: Etiological Insights and Degradation Strategies. Int. J. Mol. Sci..

[64] Graff-Radford J., Yong K.X.X., Apostolova L.G., Bouwman F.H., Carrillo M., Dickerson B.C., Rabinovici G.D., Schott J.M., Jones D.T., Murray M.E. (2021). New insights into atypical Alzheimer’s disease in the era of biomarkers. Lancet Neurol..

[65] Narasimhan S., Holtzman D.M., Apostolova L.G., Cruchaga C., Masters C.L., Hardy J., Villemagne V.L., Bell J., Cho M., Hampel H. (2024). Apolipoprotein E in Alzheimer’s disease trajectories and the next-generation clinical care pathway. Nat. Neurosci..

[66] Gan-Or Z. (2024). Clinical genetic testing in Parkinson’s disease should become part of routine patient care. Brain.

[67] Mouro Pinto R., Murtha R., Azevedo A., Douglas C., Kovalenko M., Ulloa J., Crescenti S., Burch Z., Oliver E., Vitalo A. (2024). Identification of genetic modifiers of Huntington’s disease somatic CAG repeat instability by in vivo CRISPR-Cas9 genome editing. bioRxiv.

[68] Nijs M., Van Damme P. (2024). The genetics of amyotrophic lateral sclerosis. Curr. Opin. Neurol..

[69] Jalaleddini K., Jakimovski D., Keshavan A., McCurdy S., Leyden K., Qureshi F., Ghoreyshi A., Bergsland N., Dwyer M.G., Ramanathan M. (2024). Proteomic signatures of physical, cognitive, and imaging outcomes in multiple sclerosis. Ann. Clin. Transl. Neurol..

[70] Stanzione F., Giangreco I., Cole J.C. (2021). Use of molecular docking computational tools in drug discovery. Prog. Med. Chem..

[71] Morris G.M., Lim-Wilby M. (2008). Molecular Docking. Methods Mol. Biol..

[72] Dias R., de Azevedo W. (2008). Molecular Docking Algorithms. Curr. Drug Targets.

[73] Musumeci D., Ullah S., Ikram A., Roviello G.N. (2022). Novel insights on nucleopeptide binding: A spectroscopic and In Silico investigation on the interaction of a thymine-bearing tetrapeptide with a homoadenine DNA. J. Mol. Liq..

[74] Roviello V., Musumeci D., Mokhir A., Roviello G.N. (2021). Evidence of protein binding by a nucleopeptide based on a thyminedecorated L-diaminopropanoic acid through CD and in silico studies. Curr. Med. Chem..

[75] Riccardi C., Meyer A., Vasseur J.-J., Cavasso D., Russo Krauss I., Paduano L., Morvan F., Montesarchio D. (2020). Design, synthesis and characterization of cyclic NU172 analogues: A biophysical and biological insight. Int. J. Mol. Sci..

[76] Guedes I.A., de Magalhães C.S., Dardenne L.E. (2013). Receptor–ligand molecular docking. Biophys. Rev..

[77] Torres P.H.M., Sodero A.C.R., Jofily P., Silva-Jr F.P. (2019). Key Topics in Molecular Docking for Drug Design. Int. J. Mol. Sci..

[78] Mukherjee P.K., Kumar V., Mal M., Houghton P.J. (2007). Acetylcholinesterase inhibitors from plants. Phytomedicine.

[79] Lushchekina S.V., Makhaeva G.F., Novichkova D.A., Zueva I.V., Kovaleva N.V., Richardson R.R. (2018). Supercomputer modeling of dual-site acetylcholinesterase (AChE) inhibition. Supercomput. Front. Innov..

[80] Rees T.M., Brimijoin S. (2003). The role of acetylcholinesterase in the pathogenesis of Alzheimer’s disease. Drugs of Today.

[81] Peitzika S.-C., Pontiki E. (2023). A Review on Recent Approaches on Molecular Docking Studies of Novel Compounds Targeting Acetylcholinesterase in Alzheimer Disease. Molecules.

[82] Ramalingam A., Sambandam S., Medimagh M., Al-Dossary O., Issaoui N., Wojcik M.J. (2021). Study of a new piperidone as an anti-Alzheimer agent: Molecular docking, electronic and intermolecular interaction investigations by DFT method. J. King Saud Univ. -Sci..

[83] Kısa D., Korkmaz N., Taslimi P., Tuzun B., Tekin Ş., Karadag A., Şen F. (2020). Bioactivity and molecular docking studies of some nickel complexes: New analogues for the treatment of Alzheimer, glaucoma and epileptic diseases. Bioorganic Chem..

[84] Gnanaraj C., Sekar M., Fuloria S., Swain S.S., Gan S.H., Chidambaram K., Rani N.N.I.M., Balan T., Stephenie S., Lum P.T. (2022). In Silico Molecular Docking Analysis of Karanjin against Alzheimer’s and Parkinson’s Diseases as a Potential Natural Lead Molecule for New Drug Design, Development and Therapy. Molecules.

[85] Ramsay R.R. (2016). Molecular aspects of monoamine oxidase B. Prog. Neuro-Psychopharmacol. Biol. Psychiatry.

[86] Thomas T. (2000). Monoamine oxidase-B inhibitors in the treatment of Alzheimers disease. Neurobiol. Aging.

[87] El Aissouq A., Bouachrine M., Ouammou A., Khalil F. (2022). Homology modeling, virtual screening, molecular docking, molecular dynamic (MD) simulation, and ADMET approaches for identification of natural anti-Parkinson agents targeting MAO-B protein. Neurosci. Lett..

[88] Stefanis L. (2012). α-Synuclein in Parkinson’s disease. Cold Spring Harb. Perspect. Med..

[89] Lücking C., Brice* A. (2000). Alpha-synuclein and Parkinson’s disease. Cell. Mol. Life Sci. CMLS.

[90] Bendor J.T., Logan T.P., Edwards R.H. (2013). The function of α-synuclein. Neuron.

[91] Rondón-Villarreal P., López W.O.C. (2020). Identification of potential natural neuroprotective molecules for Parkinson’s disease by using chemoinformatics and molecular docking. J. Mol. Graph. Model..

[92] Berry M., Juorio A., Li X.-M., Boulton A. (1996). Aromatic L-amino acid decarboxylase: A neglected and misunderstood enzyme. Neurochem. Res..

[93] Tilgmann C., Ulmanen I. (1996). Purification methods of mammalian catechol-O-methyltransferases. J. Chromatogr. B Biomed. Sci. Appl..

[94] Formica J., Regelson W. (1995). Review of the biology of quercetin and related bioflavonoids. Food Chem. Toxicol..

[95] Ricci A., Roviello G.N. (2023). Exploring the Protective Effect of Food Drugs against Viral Diseases: Interaction of Functional Food Ingredients and SARS-CoV-2, Influenza Virus, and HSV. Life.

[96] Roviello V., Gilhen-Baker M., Vicidomini C., Roviello G.N. (2022). The healing power of clean rivers: In silico evaluation of the antipsoriatic potential of apiin and hyperoside plant metabolites contained in river waters. Int. J. Environ. Res. Public Health.

[97] Boyina H.K., Geethakhrishnan S.L., Panuganti S., Gangarapu K., Devarakonda K.P., Bakshi V., Guggilla S.R. (2020). In Silico and In Vivo Studies on Quercetin as Potential Anti-Parkinson Agent. Adv Exp Med Biol.

[98] Verma J., Khedkar V.M., Coutinho E.C. (2010). 3D-QSAR in drug design-a review. Curr. Top. Med. Chem..

[99] El Fadili M., Er-rajy M., Imtara H., Kara M., Zarougui S., Altwaijry N., Al Kamaly O., Al Sfouk A., Elhallaoui M. (2022). 3D-QSAR, ADME-Tox In Silico Prediction and Molecular Docking Studies for Modeling the Analgesic Activity against Neuropathic Pain of Novel NR2B-Selective NMDA Receptor Antagonists. Processes.

[100] Wolfe G.I., Barohn R.J. (2002). Painful peripheral neuropathy. Curr. Treat. Options Neurol..

[101] Marchettini P., Lacerenza M., Mauri E., Marangoni C. (2006). Painful peripheral neuropathies. Curr. Neuropharmacol..

[102] Furgała-Wojas A., Kowalska M., Nowaczyk A., Fijałkowski Ł., Sałat K. (2020). Comparison of Bromhexine and its Active Metabolite—Ambroxol as Potential Analgesics Reducing Oxaliplatin-induced Neuropathic Pain—Pharmacodynamic and Molecular Docking Studies. Curr. Drug Metab..

[103] Mann J.D. (1979). Production of solasodine for the pharmaceutical industry. Adv. Agron..

[104] Verma S., Kuhad A., Bhandari R., Prasad S.K., Shakya A., Prasad R.S., Sinha S.K. (2020). Effect of ethanolic extract of Solanum virginianum Linn. on neuropathic pain using chronic constriction injury rat model and molecular docking studies. Naunyn-Schmiedeberg’s Arch. Pharmacol..

[105] Stephenson N., Shane E., Chase J., Rowland J., Ries D., Justice N., Zhang J., Chan L., Cao R. (2019). Survey of Machine Learning Techniques in Drug Discovery. Curr. Drug Metab..

[106] Sun M., Han T.X., Ming-Chang L., Khodayari-Rostamabad A. Multiple Instance Learning Convolutional Neural Networks for object recognition. Proceedings of the 2016 23rd International Conference on Pattern Recognition (ICPR).

[107] Tăuţan A.-M., Ionescu B., Santarnecchi E. (2021). Artificial intelligence in neurodegenerative diseases: A review of available tools with a focus on machine learning techniques. Artif. Intell. Med..

[108] Spencer M., Eickholt J., Cheng J. (2015). A Deep Learning Network Approach to ab initio Protein Secondary Structure Prediction. IEEE/ACM Trans. Comput. Biol. Bioinform..

[109] Li H., Hou J., Adhikari B., Lyu Q., Cheng J. (2017). Deep learning methods for protein torsion angle prediction. BMC Bioinform..

[110] Xue L.C., Dobbs D., Bonvin A.M.J.J., Honavar V. (2015). Computational prediction of protein interfaces: A review of data driven methods. FEBS Lett..

[111] Taha K. (2023). Semi-supervised and un-supervised clustering: A review and experimental evaluation. Inf. Syst..

[112] Xia Z., Wu L.-Y., Zhou X., Wong S.T.C. (2010). Semi-supervised drug-protein interaction prediction from heterogeneous biological spaces. BMC Syst. Biol..

[113] Wang C., Kurgan L. (2020). Survey of Similarity-Based Prediction of Drug-Protein Interactions. Curr. Med. Chem..

[114] Zhou H., Gao M., Skolnick J. (2015). Comprehensive prediction of drug-protein interactions and side effects for the human proteome. Sci. Rep..

[115] Gunther E.C., Stone D.J., Gerwien R.W., Bento P., Heyes M.P. (2003). Prediction of clinical drug efficacy by classification of drug-induced genomic expression profiles in vitro. Proc. Natl. Acad. Sci. USA.

[116] Myszczynska M.A., Ojamies P.N., Lacoste A.M.B., Neil D., Saffari A., Mead R., Hautbergue G.M., Holbrook J.D., Ferraiuolo L. (2020). Applications of machine learning to diagnosis and treatment of neurodegenerative diseases. Nat. Rev. Neurol..

[117] Sistare F.D., Dieterle F., Troth S., Holder D.J., Gerhold D., Andrews-Cleavenger D., Baer W., Betton G., Bounous D., Carl K. (2010). Towards consensus practices to qualify safety biomarkers for use in early drug development. Nat. Biotechnol..

[118] Rolan P., Danhof M., Stanski D., Peck C. (2007). Current issues relating to drug safety especially with regard to the use of biomarkers: A meeting report and progress update. Eur. J. Pharm. Sci..

[119] Du T., Liao L., Wu C.H., Sun B. (2016). Prediction of residue-residue contact matrix for protein-protein interaction with Fisher score features and deep learning. Methods.

[120] Scott D.E., Bayly A.R., Abell C., Skidmore J. (2016). Small molecules, big targets: Drug discovery faces the protein–protein interaction challenge. Nat. Rev. Drug Discov..

[121] Santos R., Ursu O., Gaulton A., Bento A.P., Donadi R.S., Bologa C.G., Karlsson A., Al-Lazikani B., Hersey A., Oprea T.I. (2016). A comprehensive map of molecular drug targets. Nat. Rev. Drug Discov..

[122] Gootjes-Dreesbach L., Sood M., Sahay A., Hofmann-Apitius M., Fröhlich H. (2020). Variational Autoencoder Modular Bayesian Networks for Simulation of Heterogeneous Clinical Study Data. Front. Big Data.

[123] Goetz C.G., Tilley B.C., Shaftman S.R., Stebbins G.T., Fahn S., Martinez-Martin P., Poewe W., Sampaio C., Stern M.B., Dodel R. (2008). Movement Disorder Society-sponsored revision of the Unified Parkinson’s Disease Rating Scale (MDS-UPDRS): Scale presentation and clinimetric testing results. Mov. Disord..

[124] Wolpert D.H. (1996). The Lack of A Priori Distinctions Between Learning Algorithms. Neural Comput..

[125] Dara S., Dhamercherla S., Jadav S.S., Babu C.H.M., Ahsan M.J. (2021). Machine Learning in Drug Discovery: A Review. Artif. Intell. Rev..

[126] Cervantes J., Garcia-Lamont F., Rodríguez-Mazahua L., Lopez A. (2020). A comprehensive survey on support vector machine classification: Applications, challenges and trends. Neurocomputing.

[127] Matsumoto A., Aoki S., Ohwada H. (2016). Comparison of Random Forest and SVM for Raw Data in Drug Discovery: Prediction of Radiation Protection and Toxicity Case Study. Int. J. Mach. Learn. Comput..

[128] de Brevern A.G., Wang Q., Feng Y., Huang J., Wang T., Cheng G. (2017). A novel framework for the identification of drug target proteins: Combining stacked auto-encoders with a biased support vector machine. PLoS ONE.

[129] Huang S., Cai N., Pacheco P.P., Narrandes S., Wang Y., Xu W. (2018). Applications of support vector machine (SVM) learning in cancer genomics. Cancer Genom. Proteom..

[130] Maltarollo V.G., Kronenberger T., Espinoza G.Z., Oliveira P.R., Honorio K.M. (2018). Advances with support vector machines for novel drug discovery. Expert Opin. Drug Discov..

[131] Hastie T., Tibshirani R., Friedman J. (2009). The Elements of Statistical Learning.

[132] Cano G., Garcia-Rodriguez J., Garcia-Garcia A., Perez-Sanchez H., Benediktsson J.A., Thapa A., Barr A. (2017). Automatic selection of molecular descriptors using random forest: Application to drug discovery. Expert Syst. Appl..

[133] Rahman R., Matlock K., Ghosh S., Pal R. (2017). Heterogeneity Aware Random Forest for Drug Sensitivity Prediction. Sci. Rep..

[134] Samigulina G., Zarina S. (2017). Immune Network Technology on the Basis of Random Forest Algorithm for Computer-Aided Drug Design. Bioinformatics and Biomedical Engineering. IWBBIO.

[135] Lenhof K., Eckhart L., Gerstner N., Kehl T., Lenhof H.-P. (2022). Simultaneous regression and classification for drug sensitivity prediction using an advanced random forest method. Sci. Rep..

[136] Rumelhart D.E., Hinton G.E., Williams R.J. (1986). Learning representations by back-propagating errors. Nature.

[137] Honório K.M., De Lima E.F., Quiles M.G., Romero R.A.F., Molfetta F.A., Da Silva A.B.F. (2010). Artificial Neural Networks and the Study of the Psychoactivity of Cannabinoid Compounds. Chem. Biol. Drug Des..

[138] de Molfetta F.A., Angelotti W.F.D., Romero R.A.F., Montanari C.A., da Silva A.B.F. (2008). A neural networks study of quinone compounds with trypanocidal activity. J. Mol. Model..

[139] Scotti L., Ishiki H., Mendonça Júnior F., da Silva M., Scotti M. (2015). Artificial Neural Network Methods Applied to Drug Discovery for Neglected Diseases. Comb. Chem. High Throughput Screen..

[140] Stokes A., Hum W., Zaslavsky J. (2021). A minimal-input multilayer perceptron for predicting drug-drug interactions without knowledge of drug structure. STEM Fellowsh. J..

[141] LeCun Y., Bengio Y., Hinton G. (2015). Deep learning. Nature.

[142] Carkli Yavuz B., Yurtay N., Ozkan O. (2018). Prediction of Protein Secondary Structure with Clonal Selection Algorithm and Multilayer Perceptron. IEEE Access.

[143] Gómez-Bombarelli R., Wei J.N., Duvenaud D., Hernández-Lobato J.M., Sánchez-Lengeling B., Sheberla D., Aguilera-Iparraguirre J., Hirzel T.D., Adams R.P., Aspuru-Guzik A. (2018). Automatic Chemical Design Using a Data-Driven Continuous Representation of Molecules. ACS Cent. Sci..

[144] Xiong Z., Cheng Z., Lin X., Xu C., Liu X., Wang D., Luo X., Zhang Y., Jiang H., Qiao N. (2021). Facing small and biased data dilemma in drug discovery with enhanced federated learning approaches. Sci. China Life Sci..

[145] Martinelli D.D. (2022). Generative machine learning for de novo drug discovery: A systematic review. Comput. Biol. Med..

[146] Kashyap K., Siddiqi M.I. (2021). Recent trends in artificial intelligence-driven identification and development of anti-neurodegenerative therapeutic agents. Mol. Divers..

[147] Moret M., Pachon Angona I., Cotos L., Yan S., Atz K., Brunner C., Baumgartner M., Grisoni F., Schneider G. (2023). Leveraging molecular structure and bioactivity with chemical language models for de novo drug design. Nat. Commun..

[148] Hansch C., Fujita T. (2002). p-σ-π Analysis. A Method for the Correlation of Biological Activity and Chemical Structure. J. Am. Chem. Soc..

[149] Cherkasov A., Muratov E.N., Fourches D., Varnek A., Baskin I.I., Cronin M., Dearden J., Gramatica P., Martin Y.C., Todeschini R. (2014). QSAR Modeling: Where Have You Been? Where Are You Going To?. J. Med. Chem..

[150] Mitchell J.B.O. (2014). Machine learning methods in chemoinformatics. WIREs Comput. Mol. Sci..

[151] Buckner F.S., Ekins S., Lage de Siqueira-Neto J., McCall L.-I., Sarker M., Yadav M., Ponder E.L., Kallel E.A., Kellar D., Chen S. (2015). Machine Learning Models and Pathway Genome Data Base for Trypanosoma cruzi Drug Discovery. PLoS Neglected Trop. Dis..

[152] Neves B.J., Braga R.C., Melo-Filho C.C., Moreira-Filho J.T., Muratov E.N., Andrade C.H. (2018). QSAR-Based Virtual Screening: Advances and Applications in Drug Discovery. Front. Pharmacol..

[153] Goodman W.K., Grice D.E., Lapidus K.A.B., Coffey B.J. (2014). Obsessive-Compulsive Disorder. Psychiatr. Clin. N. Am..

[154] Farghali H., Kutinová Canová N., Arora M. (2021). The potential applications of artificial intelligence in drug discovery and development. Physiol. Res..

[155] Subramanian G., Ramsundar B., Pande V., Denny R.A. (2016). Computational Modeling of β-Secretase 1 (BACE-1) Inhibitors Using Ligand Based Approaches. J. Chem. Inf. Model..

[156] Vatansever S., Schlessinger A., Wacker D., Kaniskan H.Ü., Jin J., Zhou M.M., Zhang B. (2021). Artificial intelligence and machine learning-aided drug discovery in central nervous system diseases: State-of-the-arts and future directions. Med. Res Rev..

[157] Sharma A., Lysenko A., Jia S., Boroevich K.A., Tsunoda T. (2024). Advances in AI and machine learning for predictive medicine. J Hum Genet..

[158] Panizza E., Cerione R.A. (2024). An interpretable deep learning framework identifies proteomic drivers of Alzheimer’s disease. Front. Cell Dev. Biol..

[159] Blanco K., Salcidua S., Orellana P., Sauma-Pérez T., León T., Steinmetz L.C.L., Ibañez A., Duran-Aniotz C., de la Cruz R. (2023). Systematic review: Fluid biomarkers and machine learning methods to improve the diagnosis from mild cognitive impairment to Alzheimer’s disease. Alzheimers Res. Ther..

[160] Wang X., Ye T., Jiang D., Zhou W., Zhang J. (2024). Alzheimer’s Disease Neuroimaging Initiative. Characterizing the clinical heterogeneity of early symptomatic Alzheimer’s disease: A data-driven machine learning approach. Front. Aging Neurosci..

[161] Park I., Lee S.K., Choi H.C., Ahn M.E., Ryu O.H., Jang D., Lee U., Kim Y.J. (2024). Machine Learning Model for Mild Cognitive Impairment Stage Based on Gait and MRI Images. Brain Sci..

[162] Baggett D.W., Nath A. (2018). The Rational Discovery of a Tau Aggregation Inhibitor. Biochemistry.

[163] Hernandez J.J., Pryszlak M., Smith L., Yanchus C., Kurji N., Shahani V.M., Molinski S.V. (2017). Giving Drugs a Second Chance: Overcoming Regulatory and Financial Hurdles in Repurposing Approved Drugs as Cancer Therapeutics. Front Oncol..

[164] Rodriguez S., Hug C., Todorov P., Moret N., Boswell S.A., Evans K., Zhou G., Johnson N.T., Hyman B.T., Sorger P.K. (2021). Machine learning identifies candidates for drug repurposing in Alzheimer’s disease. Nat. Commun..

[165] Oliveros G., Wallace C.H., Chaudry O., Liu Q., Qiu Y., Xie L., Rockwell P., Figueiredo-Pereira M.E., Serrano P.A. (2023). Repurposing ibudilast to mitigate Alzheimer’s disease by targeting inflammation. Brain.

[166] Angelopoulou E., Pyrgelis E.S., Piperi C. (2022). Emerging potential of the phosphodiesterase (PDE) inhibitor ibudilast for neurodegenerative diseases: An update on preclinical and clinical evidence. Molecules.

[167] Rolan P., Hutchinson M., Johnson K. (2009). Ibudilast: A review of its pharmacology, efficacy, and safety in respiratory and neurological disease. Expert Opin. Pharmacother..

[168] Abdul Manap A.S., Almadodi R., Sultana S., Sebastian M.G., Kavani K.S., Lyenouq V.E., Shankar A. (2024). Alzheimer’s disease: A review on the current trends of the effective diagnosis and therapeutics. Front. Aging Neurosci..

[169] Kim A.Y., Al Jerdi S., MacDonald R., Triggle C.R. (2024). Alzheimer’s disease and its treatment—Yesterday, today, and tomorrow. Front. Pharmacol..

[170] Zhang J., Zhang Y., Wang J., Xia Y., Zhang J., Chen L. (2024). Recent advances in Alzheimer’s disease: Mechanisms, clinical trials, and new drug development strategies. Signal Transduct. Target Ther..

[171] Sequeira L., Benfeito S., Fernandes C., Lima I., Peixoto J., Alves C., Machado C.S., Gaspar A., Borges F., Chavarria D. (2024). Drug development for Alzheimer’s and Parkinson’s disease: Where do we go now?. Pharmaceutics.

[172] Kang C. (2024). Donanemab: First approval. Drugs.

[173] Martins A.C., Oshiro M.Y., Albericio F., de la Torre B.G. (2024). Food and Drug Administration (FDA) approvals of biological drugs in 2023. Biomedicines.

[174] Xiong B., Song Z., Wang L., Zhang A., Zhou Y., Zheng N., Wei Y., Chen Y., Sun H. (2024). Can targeted protein degradation technology provide a potential breakthrough in the development of anti-AD drugs?. ACS Chem. Neurosci..

[175] Yajing M., Sufang L., Qingfeng Z., Zhonghua L., Zhijian Z., Bin Y. (2024). Approved drugs and natural products at clinical stages for treating Alzheimer’s disease. Chin. J. Nat. Med..

[176] Jin M., Noble J.M. (2024). What’s in it for me? Contextualizing the potential clinical impacts of lecanemab, donanemab, and other anti-β-amyloid monoclonal antibodies in early Alzheimer’s disease. eNeuro.

[177] Musiek E.S., McDade E., Holtzman D.M. (2023). Lecanemab ushers in a new era of anti-amyloid therapy for Alzheimer’s disease. Ann. Neurol..

[178] Cohen S., van Dyck C.H., Gee M., Doherty T., Kanekiyo M., Dhadda S., Li D., Hersch S., Irizarry M., Kramer L.D. (2023). Lecanemab Clarity AD: Quality-of-life results from a randomized, double-blind phase 3 trial in early Alzheimer’s disease. J. Prev. Alzheimers Dis..

[179] Honig L.S., Barakos J., Dhadda S., Kanekiyo M., Reyderman L., Irizarry M., Kramer L.D., Swanson C.J., Sabbagh M. (2023). ARIA in patients treated with lecanemab (BAN2401) in a phase 2 study in early Alzheimer’s disease. Alzheimers Dement..

[180] Shcherbinin S., Evans C.D., Lu M., Andersen S.W., Pontecorvo M.J., Willis B.A., Gueorguieva I., Hauck P.M., Brooks D.A., Mintun M.A. (2022). Association of amyloid reduction after donanemab treatment with tau pathology and clinical outcomes: The TRAILBLAZER-ALZ randomized clinical trial. JAMA Neurol..

[181] Sims J.R., Zimmer J.A., Evans C.D., Lu M., Ardayfio P., Sparks J., Wessels A.M., Shcherbinin S., Wang H., Monkul Nery E.S. (2023). Donanemab in early symptomatic Alzheimer disease: The TRAILBLAZER-ALZ 2 randomized clinical trial. JAMA.

[182] Mintun M.A., Lo A.C., Duggan Evans C., Wessels A.M., Ardayfio P.A., Andersen S.W., Shcherbinin S., Sparks J., Sims J.R., Brys M. (2021). Donanemab in early Alzheimer’s disease. N. Engl. J. Med..

